# Highly Sensitive Heterojunction‐Gated Phototransistor With Detection Wavelength Ranged From 350 to 1700 Nm

**DOI:** 10.1002/advs.202522627

**Published:** 2026-01-12

**Authors:** Hongkun Duan, Wenyu Zhang, Tao Luo, XiaoLu Xia, Qianxi Yang, Ying Yan, Shengmei Gao, Xitian Yin, Yixiao Niu, Zhiyu Zhao, Jianbing Zhang, Haobin He, Jiang Tang, Ying Wang, Zhiyong Zhang

**Affiliations:** ^1^ School of Integrated Circuits Beijing University of Posts and Telecommunications Beijing China; ^2^ Key Laboratory of Luminescence & Optical Information School of Physical Science and Engineering Ministry of Education Beijing Jiaotong University Beijing China; ^3^ School of Integrated Circuits Huazhong University of Science and Technology Hubei China; ^4^ Key Laboratory for the Physics and Chemistry of Nanodevices and Center for Carbon‐based Electronics School of Electronics Peking University Beijing China; ^5^ Chongqing Institute of Carbon‐Based Integrated Circuits Peking University Chongqing China; ^6^ Wuhan National Laboratory For Optoelectronics (WNLO) Huazhong University of Science and Technology Wuhan China

**Keywords:** carbon nanotube, field‐effect transistor, phototransistor, semiconductor quantum dots, short‐wave infrared detection

## Abstract

Sensitive photodetection covering UV, visible, and short‐wave infrared (SWIR) lights will greatly promote applications in all‐weather surveillance, remote sensing, and non‐destructive inspection, but remains challenging in terms of bandwidth or dark noise based on either conventional semiconductors or emerging low‐dimensional materials. Here, we take full advantage of the excellent designability and compatibility of the heterojunction‐gated field‐effect transistor (HGFET) phototransistor, and extend the SWIR detection upper limit from 1400 to 1700 nm through optimizing the lead sulfide (PbS) colloidal quantum dots (CQDs) based diode on the gate. Specifically, the mean diameter of CQDs is increased from 3.8 to 6.0 nm to enable efficient long‐wavelength (1700 nm) absorption, and a hybrid ligand passivation strategy is used to significantly suppress defect states on the nonpolar (100) facets, thereby enhancing heterojunction photovoltage. The resulting HGFETs exhibit a broadband radiation detection from 350 to 1700 nm with a room‐temperature detectivity of up to 5.7 × 10^13 ^cm Hz^1/2^ W^−1^ and a minimum detectable power density of 6.4 nW cm^−2^ at 1650 nm. The hybrid‐passivated CQD HGFETs provide a possible route toward next‐generation, highly sensitive, and broadband infrared photodetectors from UV to short‐wave infrared (beyond 1700 nm) light.

## Graphical abstract

This work demonstrates a heterojunction‐gated infrared phototransistor for broadband detection from 350 to 1700 nm. By suppressing defect states on nonpolar (100) facets of large PbS quantum dots via hybrid ligand passivation, the device achieves a room‐temperature detectivity of 5.7 × 10^13^ Jones at 1650 nm. Wafer‐scale fabrication highlights its potential for highly integrated SWIR imaging systems.

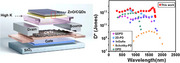

## 1 Introduction

Broadband detection of optical radiation covering UV, visible, and short‐wave infrared (SWIR) regions beyond 1100 nm is critical for a wide range of applications, including all‐weather surveillance, autonomous driving, remote sensing, industrial inspection, and machine vision [1, 2, 3]. Compared to using two or multiple detectors, a single broadband detector covering these three wavebands offers significant advantages in imaging systems. First, the multi‐in‐one detector eliminates the need for optical splicing, registration, and calibration across multiple discrete detectors, thereby significantly simplifying the optical system architecture and reducing its size, weight, power consumption, and cost [4, 5, 6]. Second, it avoids errors associated with multi‐sensor data fusion. Furthermore, it ensures strictly simultaneous and co‐registered acquisition of signals across different spectral bands, which is essential for analyzing transient phenomena and eliminating motion blur [7, 8]. However, existing single detectors cannot meet the requirements for high‐sensitivity detection. Silicon‐based visible‐light detectors such as CCD or CMOS can be extended into the UV and near‐infrared, but their infrared detection wavelength remains below 1100 nm [9, 10]. InGaAs SWIR detectors suffer from a rapid decline in sensitivity in the visible range due to the absorption limitations of the InP substrate [3, 11]. Recently, broadband photodetectors based on various 2D materials such as graphene have been extensively reported, but most of them suffered from poor sensitivity owing to the low absorption coefficients of their ultra‐thin bodies [12, 13, 14, 15, 16].

The emerging low‐dimensional semiconductors make the design and construction of photodetectors more flexible and provide the possibility of building devices that significantly surpass previous ones. As a typical example, a heterojunction‐gated field‐effect transistor (HGFET), comprising a colloidal quantum dot (CQD) heterojunction and a carbon nanotube (CNT) film FET isolated by a high‐κ gate dielectric, has been developed based on an opto‐electric decoupling mechanism to achieve high inherent gain and record‐level specific detectivity [17, 18, 19]. Furthermore, the absorption wavelength and bandwidth of HGFET are designable by adjusting the semiconducting quantum dots, enabling the possible realization of a multi‐in‐one sensitive detector covering UV, visible, and SWIR regions beyond 1100 nm. However, the reported HGFET typically exhibited a detection wavelength cut‐off around 1400 nm, mainly due to the light absorption limit arising from the large bandgap of the CQDs, and thus fail to cover 1550 nm, which is an important atmospheric transmission window [3, 20] for numerous applications such as LiDAR, optical communication, and night vision [21, 22, 23, 24]. The inability of current detectors to cover this band significantly undermines the universality of such broadband photodetectors. It is therefore very important to extend the cut‐off wavelength of HGFET to approximately 1700 nm, which, however, faces challenges related to preparing CQDs with larger diameters and constructing a high‐quality p‐i‐n junction. With increasing CQD size, the surface termination of semiconducting CQDs typically transitions from polar (111) facets to non‐polar (100) facets, which possess a significantly higher density of defects acting as non‐radiative recombination centers that quench photogenerated carriers, thereby causing a drastic reduction in both photovoltage and photoconversion efficiency [25, 26, 27]. Therefore, to extend the detection wavelength to 1700 nm in HGFETs by increasing the CQD size, it is necessary to develop an effective passivation strategy and construct a diode with a high enough photovoltage.

In this work, we increase the mean diameter of CQDs from 3.8 to 6.0 nm to enable efficient long‐wavelength (1700 nm) absorption and develop a hybrid ligand passivation strategy that significantly suppresses defect states on nonpolar (100) facets, enhancing the heterojunction photovoltage from 0.16 to 0.23 V. We then integrate the CQD‐based diode with the CNT‐film‐based HGFET, which exhibits broadband radiation detection from 350 to 1700 nm, with a room‐temperature detectivity up to 5.7 × 10^13 ^cm Hz^1/2^ W^−1^ and a minimum detectable power density of 6.4 nW cm^−2^ at 1650 nm. The hybrid‐passivated CQD HGFETs thus provide a possible route toward next‐generation, highly sensitive, and broadband infrared photodetectors operating from UV to short‐wave infrared (beyond 1700 nm) light.

## 2 Results and Discussion

### 2.1 Synthesis and Surface Passivation of PbS CQDs

**FIGURE 1:**
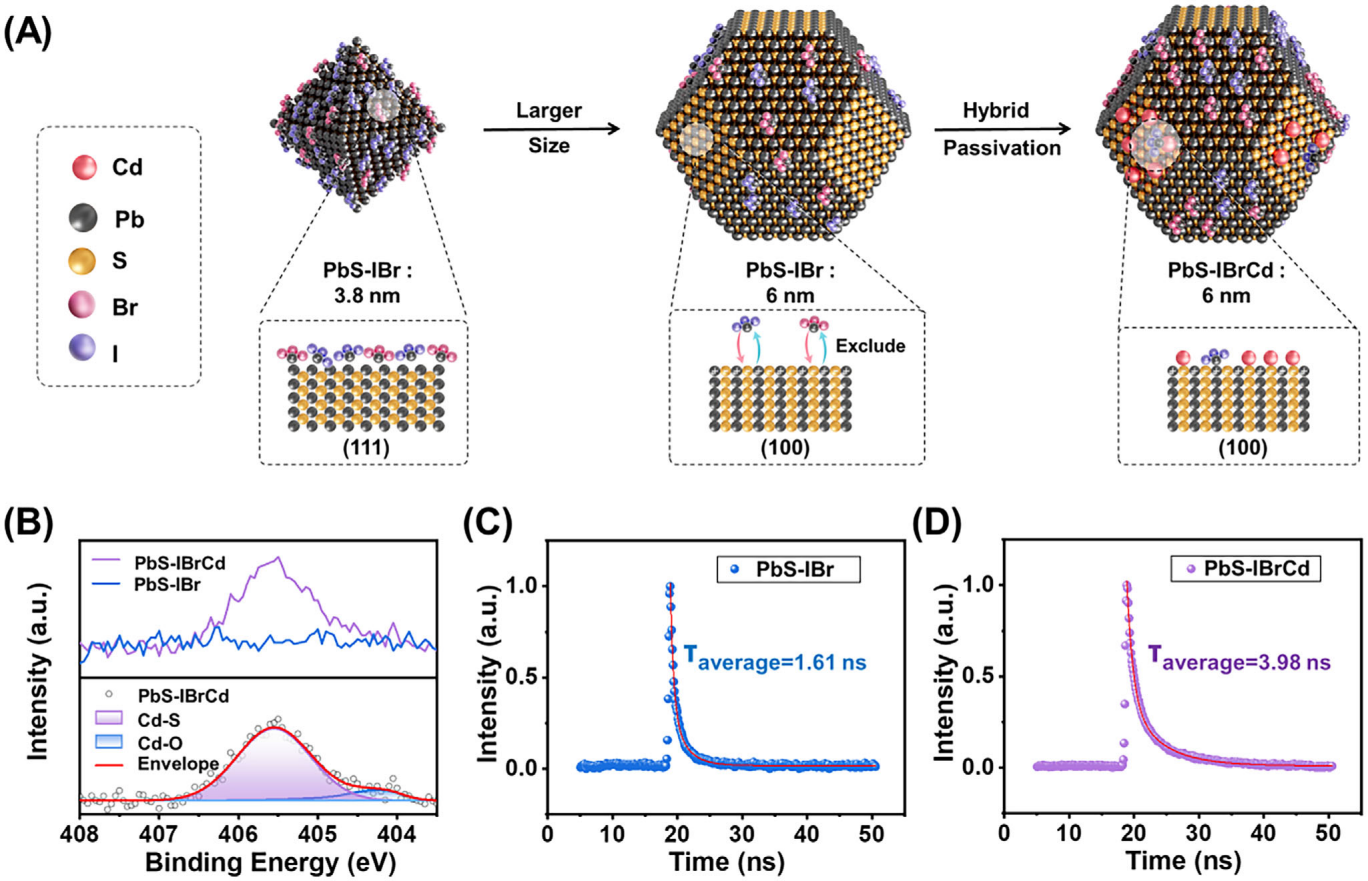
Surface facets and optical characterization of synthesized PbS CQDs. (A) Schematic illustrations showing the morphology and exposed surface facets of 3.8 nm PbS‐IBr, 6 nm PbS‐IBr, and 6 nm PbS‐IBrCd CQDs. (B) Cd 3d XPS spectra of the PbS‐IBr and PbS‐IBrCd CQD films. The Cd 3d peaks of the PbS‐IBrCd sample can be divided into two peaks associated with Cd─S and Cd─O bonds (bottom panel in A). (C) PL decay of PbS‐IBr CQD solid films. (D) PL decay of PbS‐IBrCd CQD solid films.

PbS CQDs with well‐controlled size distributions were synthesized using a refined cation‐exchange method (see Experimental Section) [27]. The exposure of specific surface facets was tuned by controlling the growth temperature and monomer concentration, balancing kinetic and thermodynamic factors during nucleation and growth. Small‐sized CQDs (∼3.8 nm, absorption peak ∼1300 nm, Figure S1), synthesized under kinetically dominated conditions, predominantly expose Pb‐rich (111) facets and adopt octahedral morphologies (Figure 1A). These polar facets are readily passivated via short‐chain halides, which provide electrostatic stabilization while minimizing interparticle spacing. By contrast, larger CQDs (∼6 nm, peak ∼1650 nm), grown under thermodynamically dominated conditions, evolve toward nearly spherical shapes rather than the cube‐like morphologies observed in kinetically controlled growth. This leads to reduced but still appreciable exposure of (100) facets composed of alternating Pb^2+^ and S^2−^ ions. These nonpolar surfaces are often insufficiently passivated, resulting in defect formation and undesired interdot fusion.

To overcome this issue, we introduced a hybrid passivation strategy leveraging the affinity of metal cations such as Na^+^ and Cd^2^^+^ for surface sulfur atoms on (100) facets [28, 29]. Specifically, halide anions (I^−^, Br^−^) were employed to passivate undercoordinated Pb^2+^ atoms, while Cd^2+^ (from CdAc_2_) selectively coordinated with S^2−^ surface sites (see Experimental Section for details). To evaluate the effectiveness of this strategy, hybrid‐passivated CQDs (PbS‐IBrCd) were compared with halide‐only controls (PbS‐IBr). Optical, scanning electron microscope (SEM), and atomic force microscopy (AFM) imaging revealed comparable surface morphology and film smoothness between PbS‐IBrCd and PbS‐IBr, indicating that hybrid passivation does not compromise film uniformity (Figure S2). High‐resolution X‐ray photoelectron spectroscopy (XPS) confirmed the exclusive presence of Cd in PbS‐IBrCd films (Figure 1B; Figure S3). The Cd 3d spectrum shows characteristic peaks at 405.6 and ∼404.3 eV, corresponding to Cd─S and Cd─O coordination [30, 31, 32], consistent with Cd^2+^ bonding to surface S^2−^ species on the CQD facets. No discernible shift is observed in the Pb 4f core‐level bonding energy upon Cd introduction, indicating that the Pb oxidation state and bulk electronic structure remain largely unaffected. In contrast, surface‐related species exhibit systematic shifts: the I 3d shift to higher binding energies, while the S 2p peak shifts to lower binding energy (Figure S3), suggesting Lewis acid‐base interactions between Cd^2+^ and surface chalcogenide/halide ligands. These interactions suppress ligand desorption and enhance passivation of undercoordinated surface sites, as reflected by the increased halide‐to‐Pb atomic ratio (Figure S3F). Time‐resolved photoluminescence (TRPL) measurements further indicated that increasing Cd additive concentration prolongs carrier lifetimes in PbS‐IBrCd films (average lifetime 3.9 ns at 0.2 mol L^−1^ CdAc_2_, Table S1) relative to PbS‐IBr control films (∼1.6 ns) (Figure 1C,D; Figure S4). Steady‐state photoluminescence (PL) spectra of both PbS‐IBr and PbS‐IBrCd films show peaks around ∼1650 nm with relatively symmetric shapes and narrow full width at half maximum (<150 nm), confirming that TRPL primarily tracks band‐edge rather than trap‐state emission (Figure S5). Together, these results demonstrate that hybrid passivation effectively mitigates surface defects, yielding higher‐quality CQD films, particularly for large‐sized CQDs with challenging surface chemistries.

### 2.2 Construction and Performance of Heterojunction Photodiodes

**FIGURE 2:**
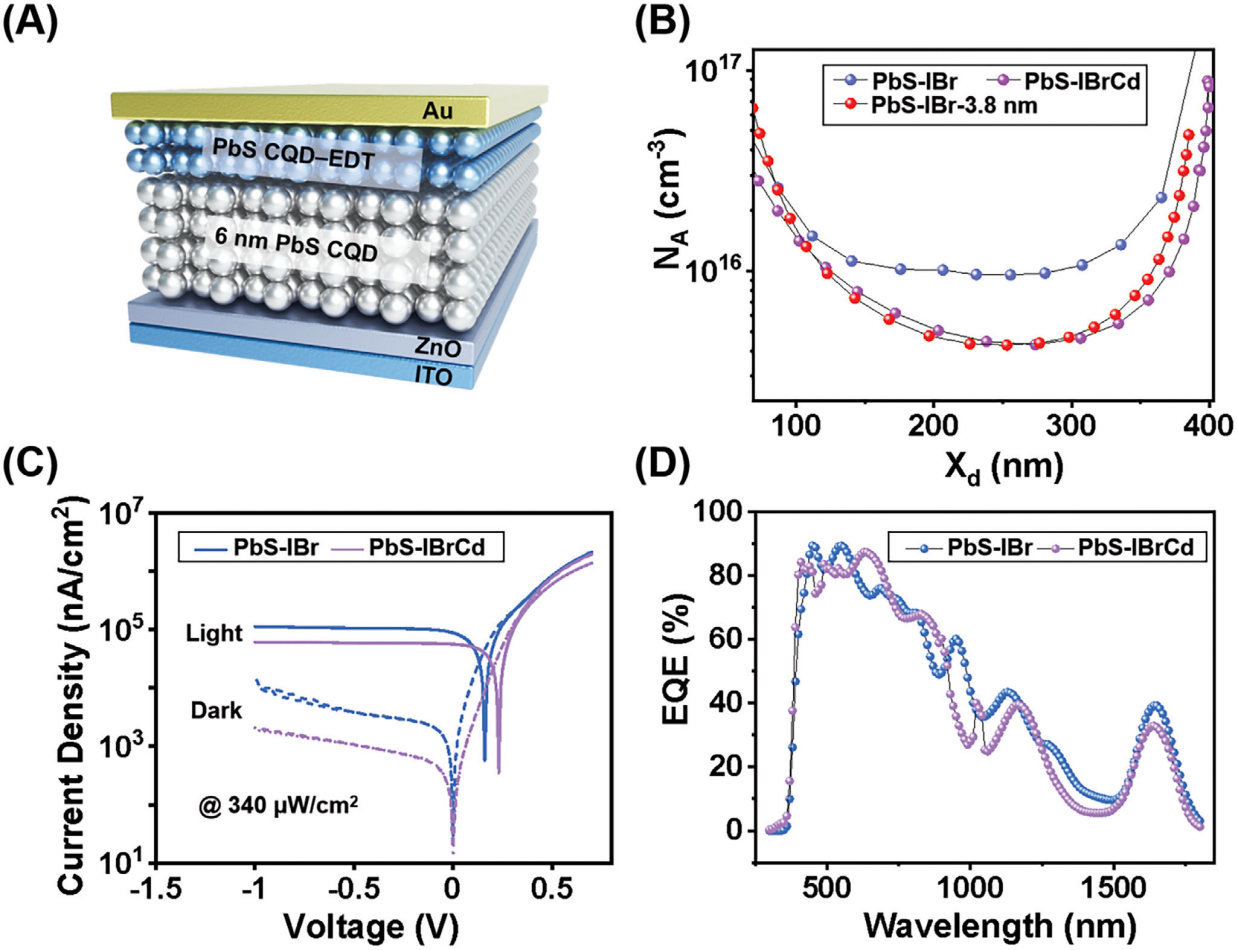
Ohmic‐contacted photodiodes for investigating the optoelectronic properties of PbS‐IBr and PbS‐IBrCd CQDs. (A) Schematic illustration of the p‐i‐n photodiode architecture. In all CQD‐based p‐i‐n heterojunction devices, the ETL and HTL consist of a 40 nm sputtered ZnO layer and a ∼30 nm EDT‐treated PbS CQD layer, respectively. (B) Defect density profiles of CQDs derived from DLCP measurements. (C) Comparison of current density‐voltage characteristics measured in the dark and under 1650 nm illumination at an intensity of 340 µW cm^−2^. (D) External quantum efficiency spectra measured at −0.1 V bias.

To investigate the optoelectronic properties and bulk trap states of the CQD films, we fabricated vertical photodiodes incorporating ohmic contacts (Figure S6). As shown in Figure 2A, the device architecture was ITO/ZnO/PbS CQD/PbS‐EDT CQD/Au, where a 40 nm sputtered ZnO electron transport layer (ETL) and a 30 nm EDT‐treated PbS CQD hole transport layer (HTL) ensured favorable band alignment. Bulk defect densities were extracted using drive‐level capacitance profiling (DLCP), which suppresses interface contributions to isolate intrinsic trap states [33, 34]. As shown in Figure 2B, hybrid passivation reduced trap density in 6 nm CQDs from ∼10^16^ cm^−3^ to 5 × 10^15^ cm^−3^, reaching values comparable to those of (111)‐terminated small CQDs.

Figure 2C compares the current density‐voltage (*J*‐*V*) characteristics of PbS‐IBrCd and PbS‐IBr devices. Hybrid passivation reduced the dark current significantly, with values of 13.5 nA cm^−2^ at 0 V bias and 0.5 µA cm^−2^ at a −0.1 V bias, corresponding to 2× and 4× reductions relative to the PbS‐IBr control device. Under 1650 nm illumination (340 µW cm^−2^), the open‐circuit photovoltage increased from 0.16 to 0.23 V with increasing Cd additives, reflecting reduced photovoltage loss (from 0.59 to 0.52 V). These improvements highlight the effectiveness of Cd passivation to suppress defect‐assisted leakage and nonradiative recombination. Notably, dark current densities across devices systematically increased with decreasing CQD bandgap—from 20 nA cm^−2^ in 970 nm devices (absorption peak ∼970 nm) [35], to 100 nA cm^−2^ in 1300 nm devices [36], and exceeding 1 µA cm^−2^ in 1650 nm devices at −0.1 V bias [37]. While residual unpassivated (100) facets may still play a role, this trend is also governed by fundamental semiconductor physics: narrower bandgap materials intrinsically exhibit higher carrier concentrations [38], which naturally lead to dark current densities.

Despite these advantages, the PbS‐IBrCd devices exhibited reduced EQE at 1650 nm (from 40 % to 33 %) and slower response speeds at higher Cd additive levels (Figure 2D; Figure S4C). The EQE data for the 3.8 nm PbS‐IBr control sample (Figure S7) confirm that the extension of detection to longer wavelengths is a direct consequence of the increased quantum dot size. This decline may stem from weakened interdot electronic coupling or band alignment shifts induced by Cd passivation [39]. Noise spectral density measurements at −0.1 V bias (Figure S8) were used to calculate specific detectivity (D*), defined as $D^{*}=R\sqrt{Af_{B}}/\sqrt{\int_{0}^{f_{B}}I_{n}^{2}\left(f\right)df}$, where *R* is responsivity, *A* is an effective device area, *I_n_* (*f*) is the frequency‐dependent noise current, and *f_B_* is the effective signal bandwidth (‐3 dB cut‐off frequency; see Experimental Section) [40]. The PbS‐IBrCd device achieved a D* of 2.2 × 10^11 ^cm Hz^1/2^ W^−1^at 1650 nm, moderately higher than the halide‐only control device (5.4 × 10^10 ^cm Hz^1/2^ W^−1^). This indicates that, although defect passivation and dark current suppression were realized, the overall sensitivity of long‐wavelength devices remains constrained by intrinsic architecture limitations.

### 2.3 Construction and Performance of CNT Film HGFETs

**FIGURE 3:**
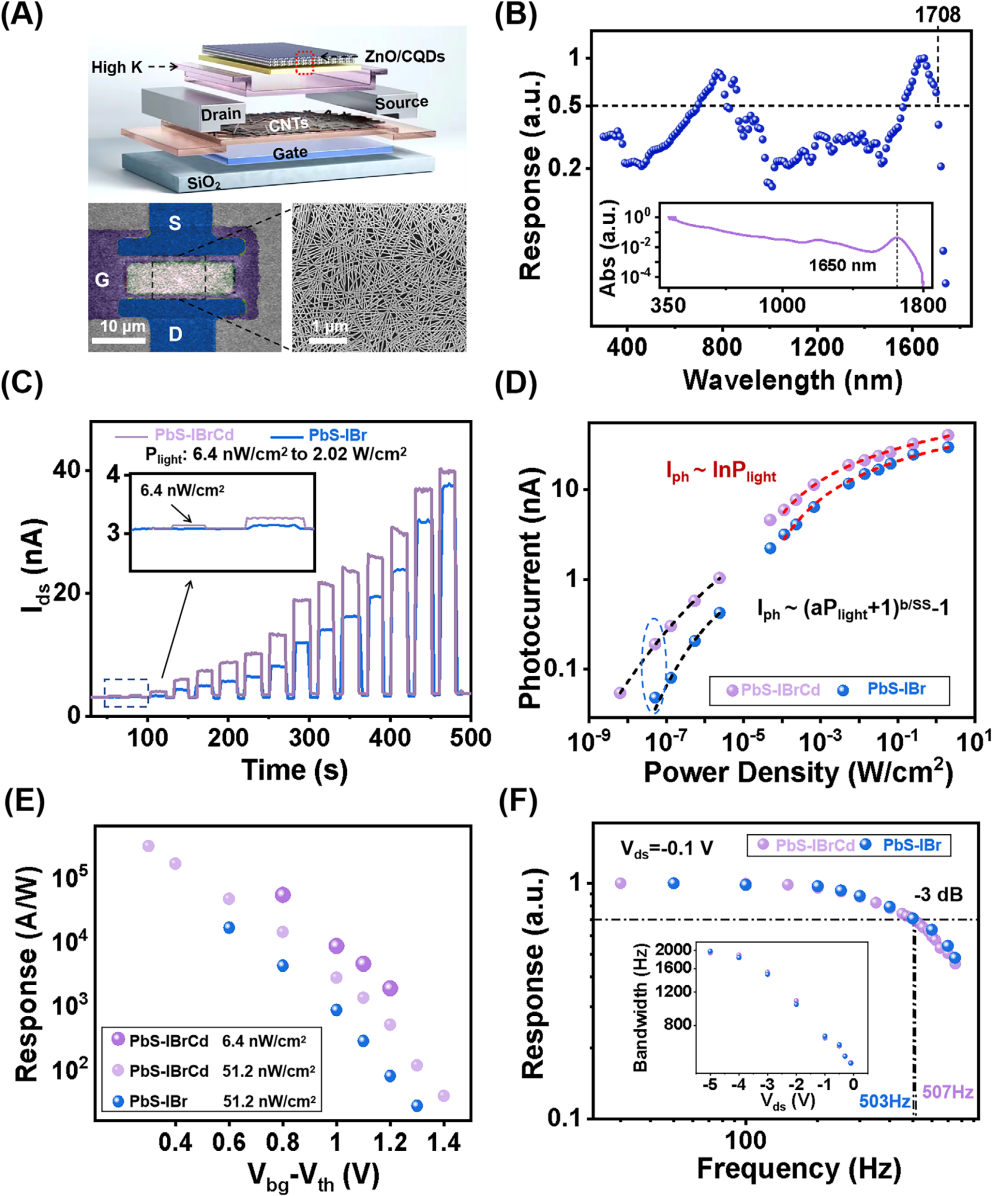
Response characteristics of HGFETs gated by PbS‐IBr and PbS‐IBrCd CQD heterojunctions. (A) Schematic diagram of an HGFET, along with an optical image of the measured device and an SEM image of the randomly oriented CNT thin film. (B) Spectral responsivity of the HGFET. Inset: absorption spectrum of the employed 6 nm‐PbS CQD thin film. (C) Time‐resolved photocurrent under 1650 nm illumination at various light intensities (6.4 nW cm^−2^ to 2.0 W cm^−2^) for typical devices biased at *V_ds_* = −0.1 V and *V_bg_* − *V_th_* = 1 V. (D) Extracted photocurrent as a function of light intensity. (E) Responsivity as a function of back‐gate bias. (F) −3 dB bandwidth of the devices. Inset: −3 dB bandwidth measured at different *V_ds_*.

To address the limitations observed in vertical photodiodes, we further developed CNT‐based HGFETs, which decouple optical response from charge readout and thereby enable independent optimization of light harvesting and charge extraction dynamics. As illustrated in Figure 3A, the back‐gated HGFET consists of a semiconducting CNT channel (10 µm width and length) capped with a conformal 6 nm high‐κ yttrium oxide (Y_2_O_3_) dielectric and a CQD heterojunction. Photovoltage generated in the CQD heterojunction modulates the CNT Fermi level and the drain–source current (*I_ds_*) via electrostatic gating. SEM images in Figure 3A show the CNT film morphology (semiconducting purity >99.9 %, density ∼35 CNTs µm^−1^) and a representative HGFET device with Pd contacts and patterned ZnO defining an 8 µm gate length. Spectral responsivity measurements (Figure 3B) show a continuous broadband photoresponse from 0.35 to 1.7 µm, with a long‐wavelength cutoff at 1708 nm. This behavior is consistent with the broadband optical absorption and the infrared absorption peak of the large‐sized CQDs (∼1658 nm; inset of Figure 3B), indicating a strong correlation between the extended photoresponse and the size‐induced redshift of optical absorption. Under 1650 nm illumination, the HGFET transfer characteristics exhibit a positive shift with increasing power density, which is attributed to the enhanced photovoltage generated at the heterojunction (Figure S9). Figure 3C compares the dynamic *I_ds_* characteristics of PbS‐IBrCd and PbS‐IBr HGFETs under 1650 nm illumination as the incident power density (*P_light_*) increases from 6.4 nW cm^−2^ to 2.0 W cm^−2^. The optical power was measured using a calibrated optical power meter (Table S2). The beam spot size was determined using an InGaAs camera operated in high‐gain mode and independently verified by the knife‐edge method (Figure S10). Measurements were taken at *V_ds_* = ‐0.1 V and *V_bg_* − *V_th_* = 1.0 V (*V_ds_*: drain–source bias,  *V_bg_*: back‐gate bias, *V_th_*: threshold voltage). The hybrid‐passivated device achieves a photocurrent (*I_ph_*) of 53.3 pA at just 6.4 nW cm^−2^ with a background current of 3.1 nA. In contrast, the PbS‐IBr control device requires approximately 51.2 nW cm^−2^ to generate a comparable photocurrent of 49.0 pA. This clearly demonstrates that hybrid passivation enhances photovoltage and reduces recombination losses. The performance advantage persists across the full spectral range due to reduced photovoltage loss (Figure S11). At low illumination levels, HGFETs operate in the subthreshold regime where the photocurrent *I_ph_* (Figure 3D) increases monotonically with *P_light_*, following a positive power‐law relationship: $I_{ph}=\left[{\left(aP_{light}+1\right)}^{\frac{b}{SS}}-1\right]I_{ds0}$, where *SS* is the subthreshold swing, *I*_*ds*0_ is the dark current, and *a* and *b* are the fitting parameters [18, 41]. This relationship can be derived from a combined PN junction and FET model, supporting the photovoltage‐induced response mechanism (see the Supporting Information and the experimental transfer curves measured in the dark and under various light intensities in Figure S9). At higher illumination intensities, the devices gradually enter the linear regime, where $I_{ph}∼lnP_{light}$ (inset of Figure S9A). This transition inherently suppresses current saturation and preserves response fidelity over a wide dynamic range.

We next investigated the *V_bg_* dependence of photoresponse to balance photogain (determined by FET transconductance, *g_m_*) against dark current (Figure S12). Figure 3E shows the extracted responsivity of both HGFET types as a function of  *V_bg_* − *V_th_*. The hybrid‐passivated device demonstrates a lower detectable *P_light_* and a responsivity exceeding 10^4^ A W^−1^, over one order of magnitude higher than the control. Importantly, frequency response measurements (Figure 3F) indicate that both devices maintain a −3 dB bandwidth of approximately 500 Hz at a low *V_ds_* of −0.1 V, confirming that Cd passivation does not compromise response speed. Transient measurements (Figure S13) further show rise and fall times of 450 and 810 µs, respectively. Upon increasing *V_ds_*, the bandwidth extends to ∼2000 Hz, attributed to the stronger lateral electric field. For  *V_ds_* beyond −4 V, the bandwidth gradually saturates, likely limited by the intrinsic photocarrier dynamics in the CQD heterojunction under open‐circuit conditions (Figure S14). Notably, the HGFET response is governed by the coupled dynamics of photovoltage buildup and channel modulation, rather than the heterojunction transit time alone, explaining the discrepancy between the effective bandwidth‐limiting time constant in the HGFET and the decay time observed in standalone CQD photodiodes (Figures S13 and S14).

**FIGURE 4:**
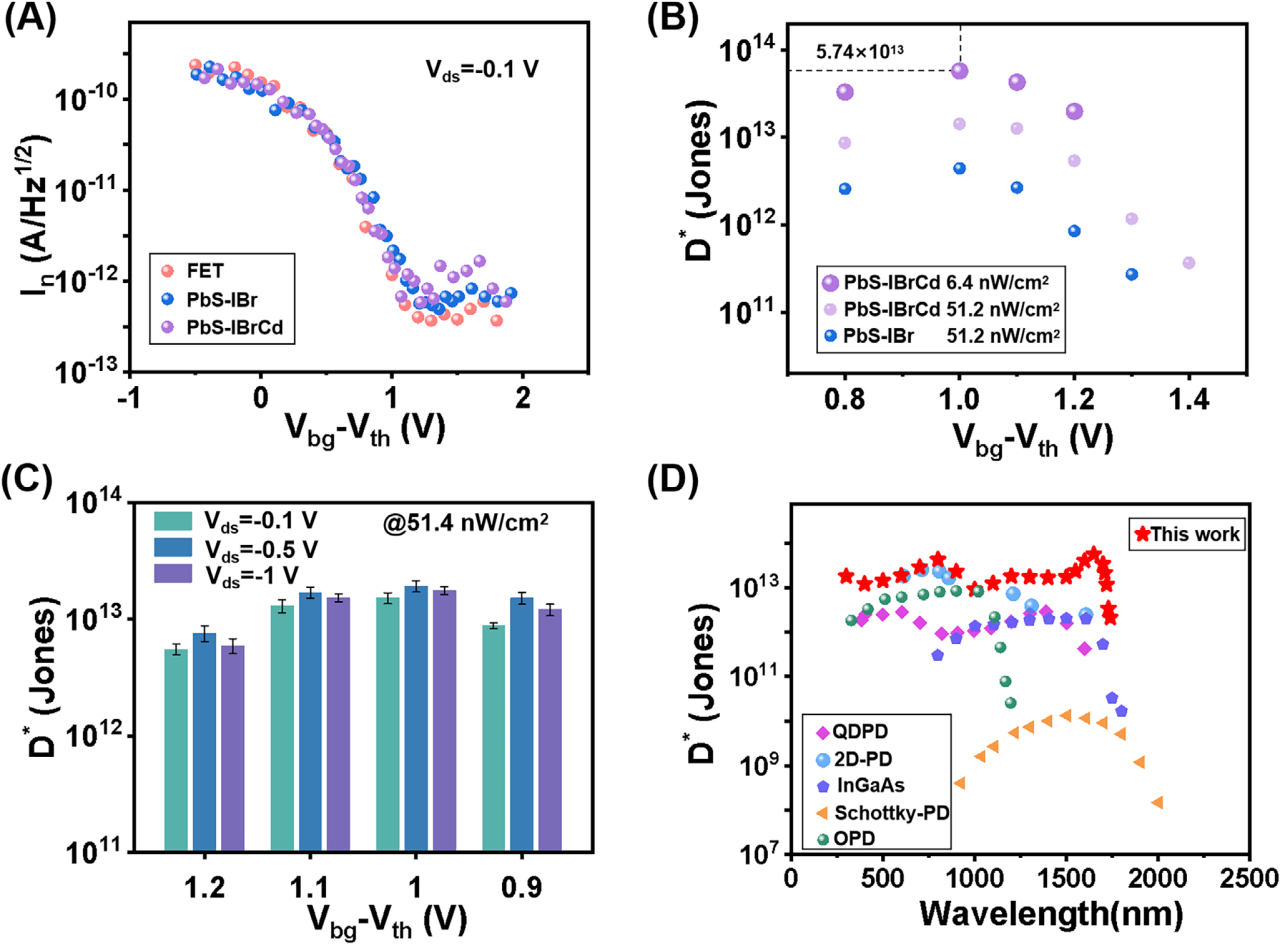
Room‐temperature specific detectivity D* for CQD HGFETs. (A) Noise current spectral density at 1 Hz for PbS‐IBrCd HGFET, PbS‐IBr HGFET and CNT FET without a heterojunction as a function of back‐gate bias. (B) Extracted D* under 1650 nm illumination at different *V_bg_* values, showing enhanced sensitivity and a lower detection limit in hybrid‐passivated devices. (C) D* as a function of drain–source bias (*V_ds_*) under various *V_bg_* values. (D) Broadband D* spectrum of the PbS‐IBrCd HGFET, compared with state‐of‐the‐art infrared photodiodes.

The frequency‐dependent current noise spectra of the PbS‐IBrCd HGFET were measured with two independent systems (PDA FS380 and Keysight E4727B) at *V_ds_* = −0.1 V (Figure S15). In the low‐frequency regime (<1 kHz), the spectra are dominated by 1/*f* noise, with current noise densities of 10^−12^–10^−13^ A Hz^−1/2^ between 1 and 100 Hz. Owing to negligible thermal noise contributions from the open‐circuit CQD heterojunction [17], the overall noise remains comparable to that of halide‐only HGFETs and bare CNT FETs across various  *V_bg_* − *V_th_* biases (Figure 4A). By combining noise spectra with the responsivity data (Figure 3E), the specific detectivity at 1650 nm was extracted and plotted as a function of  *V_bg_* (Figure 4B). The PbS‐IBrCd HGFET exhibits a peak D* of 5.7 × 10^13 ^cm Hz^1/2^ W^−1^ at  *V_bg_* − *V_th_* = 1 V, over an order of magnitude higher than the halide‐only control (4.6 × 10^12 ^cm Hz^1/2^ W^−1^) and superior to state‐of‐the‐art CQD photodiodes (typically <10^12 ^cm Hz^1/2^ W^−1^). Figure 4C shows the dependence of D* on *V_ds_* at a fixed optical power density (51.4 nW cm^−2^). While larger *V_ds_* enhances transconductance and bandwidth (Figure 3F; Figure S16), it also increases dark current and noise, resulting in nearly constant D* under a given *V_bg_* − *V_th_*. A benchmarking analysis (Figure 4D; Figure S17 and Table S3) against commercial photodiodes and reported infrared detectors [17, 19, 35, 36, 37, 42, 43, 44, 45, 46, 47, 48] highlights the superior sensitivity, broadband operation, and low‐light detection capability of the hybrid‐passivated HGFET.

### 2.4 Scalable Fabrication of HGFET Photodetectors

**FIGURE 5:**
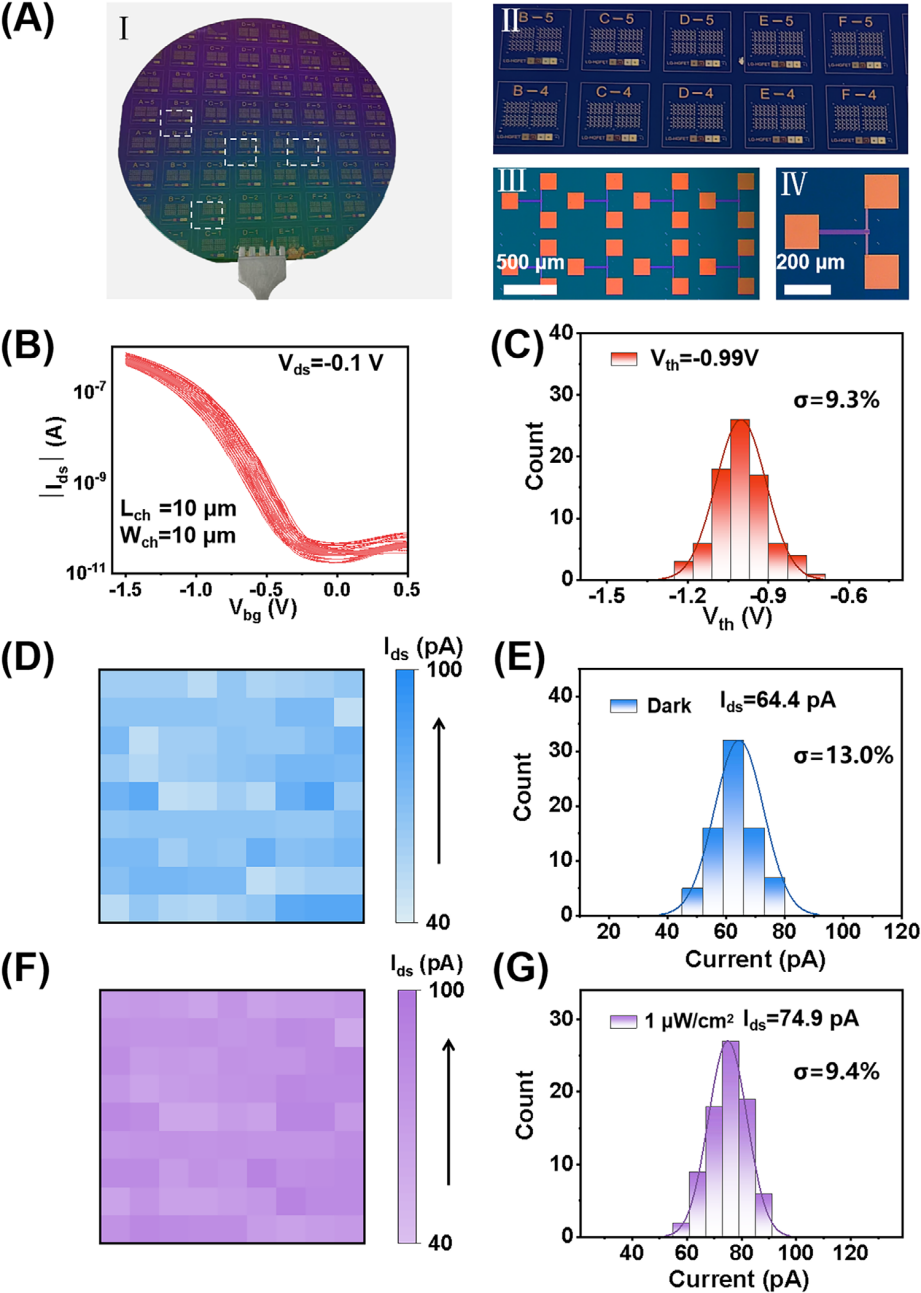
Wafer‐scale fabrication and statistical analysis of PbS CQD/CNT‐based HGFETs via a lift‐off process. (A) Photograph of a 4 inch wafer comprising 45 complete dies (I); each die contains over 80 HGFETs (II). Insets (III) and (IV) show progressively magnified optical micrographs of the device layout and an individual device, respectively. (B) Transfer curves of 81 randomly selected back‐gated CNT FETs measured at *V_ds_* = −0.1 V. (C) Statistical distribution of *V*_th_ extracted from the same 81 FETs. (D, E) Spatial map and corresponding distribution of dark current for the 81 HGFETs after deposition of CQD heterojunctions (*V_ds_* = −0.1 V and *V_bg_* = 0 V). (F, G) Spatial map and statistical distribution of photocurrent under 1650 nm illumination at an intensity of 1.0 µW cm^−2^.

Hybrid‐passivated HGFETs are highly attractive for high‐performance passive SWIR imaging due to their compatibility with large‐scale integration on CMOS platforms. To demonstrate scalability, we fabricated wafer‐scale HGFET arrays on a 4‐inch silicon substrate using standard lift‐off photolithography (Figure 5A). The processed wafer yielded 45 fully functional dies, each incorporating 84 HGFETs (Figure 5A, panels II–IV). Uniformity was first evaluated on 81 randomly selected CNT FETs prior to CQD deposition. Transfer characteristics measured at *V_ds_* = −0.1 V (Figure 5B) showed a well‐defined threshold voltage distribution centered at −0.99 V, with a coefficient of variation (CV) of 8.0 % (Figure 5C). After CQD integration, device uniformity was further assessed by mapping dark and photocurrents under 1650 nm illumination at 1.0 µW cm^−2^ (∼1 pW per device) across the wafer (Figure 5D,F). The corresponding histograms (Figure 5E,G) yielded average dark and photocurrents of 64.4 pA (CV = 13.0 %) and 74.9 pA (CV = 9.4 %), respectively, indicating a moderate increase in variability following CQD deposition. The increase in CV is mainly due to the CQD spin‐coating process. Depositing an ultrathin CQD layer (<200 nm) inherently introduces spatial thickness variations, which can locally affect p–n junction properties and device photoresponse. Additional variability may result from laboratory‐scale lift‐off fabrication, where accumulated process steps and surface irregularities can cause partial CQD detachment or local aggregation. To improve uniformity for large‐scale arrays, scalable deposition methods such as blade or slot‐die coating, dry processes in inert environments, and chemical–mechanical polishing (CMP) for substrate planarization are to be explored. In addition to scalability and device uniformity, the hybrid‐passivated HGFETs maintain stable and reproducible photoresponse over 250 on/off switching cycles under 940 nm illumination, as well as under continuous illumination for 10 min with minimal photocurrent variation. Moreover, unencapsulated devices retain over 75 % of their initial responsivity after six months of storage in a vacuum environment (vacuum level ∼1 kPa), indicating reasonable long‐term stability (Figure S18). Together, these results highlight the feasibility of wafer‐scale integration of hybrid‐ligand HGFETs for practical passive SWIR imaging applications.

## 3 Conclusions

In summary, we report a broadband and highly sensitive phototransistor based on HGFET architecture through optimizing the PbS CQDs‐based diode on the gate. The diameter of CQDs is improved from 3.8 to 6.0 nm to enable efficient long‐wavelength (1700 nm) absorption, and a hybrid ligand passivation strategy is used to significantly suppress defect states on nonpolar (100) facets for enhancing heterojunction photovoltage. The resulting HGFETs exhibit a broadband radiation detection from 350 to 1700 nm with a room‐temperature detectivity up to 5.7 × 10^13 ^cm Hz^1/2^ W^−1^ and a minimum detectable power density of 6.4 nW cm^−2^ at 1650 nm. Finally, wafer‐scale fabrication on a 4‐inch silicon substrate has been demonstrated to exhibit a preliminary uniformity (CV <15 %). Therefore, the hybrid‐passivated CQD HGFETs provide a possible route to develop a multi‐in‐one sensitive imaging system covering UV, visible, and SWIR region (beyond 1700 nm).

## 4 Experimental Section

### 4.1 Synthesis of Small‐ and Large‐Sized PbS CQDs

PbS CQDs were synthesized via a modified hot‐injection method utilizing ZnS CQDs as sacrificial sulfur precursors [27]. Briefly, ZnS CQDs were prepared by heating a mixture of zinc stearate, thioacetamide (TAA), oleylamine (OLA), and octadecene (ODE) at 140°C under nitrogen for 50 min after degassing. The product was precipitated with ethanol, collected by centrifugation, and further purified by repeated washing with n‐hexane and ethanol before being redispersed in ODE for subsequent use. For small‐sized PbS CQDs (∼3.8 nm), a Pb‐OLA precursor was first prepared by dissolving PbCl_2_ in OLA. After degassing and heating, the solution was cooled to 60°C for the rapid injection of ZnS CQDs to initiate nucleation. An additional ZnS CQD solution was then added dropwise until the excitonic absorption peak reached ∼1300 nm. The reaction was quenched in a water bath, followed by the sequential addition of n‐hexane and oleic acid. The resulting CQDs were purified via centrifugation and acetone washing. Large‐sized PbS CQDs (∼6 nm) were synthesized by maintaining the Pb‐OLA precursor at 120°C and slowly adding ZnS CQDs. The final CQDs were stored in *n*‐octane for subsequent device fabrication and characterization.

### 4.2 Passivation Strategy Through Ligand Exchange

PbS CQD inks were prepared using a phase transfer ligand exchange (PTLE) approach. A CQD dispersion was first prepared by dissolving PbS CQDs (10 mg/mL) in 10 mL of *n*‐octane. Separately, a halide source solution was obtained by dissolving 1.229 g of PbI_2_ and 0.426 g of PbBr_2_ in 10 mL of anhydrous dimethylformamide (DMF). Both solutions were filtered to ensure purity before use. The CQD dispersion was slowly injected into the halide solution (1:1 v/v) using a syringe, followed by vigorous shaking for 40 s under mild heating with a heat gun to facilitate ligand exchange. After phase separation, the upper *n*‐octane layer was removed and washed twice with an equal volume (10 mL) of fresh *n*‐octane, each time shaking for 40 s to extract unbound ligands. The final *n*‐octane layer was discarded, and the remaining CQD‐DMF phase was centrifuged at 9000 rpm for 5 min. The supernatant was removed, and the CQD pellet was vacuum‐dried to eliminate residual solvents. The resulting ligand‐exchanged CQDs were redispersed in a polar solvent mixture of DMF: Dimethyl sulfoxide: Benzotriazole: 3‐methylpyridine (DMF: DMSO: BTA: 3‐methylpyridine = 35:25:37:3, v/v) to a total volume of 1 mL.

For hybrid ligand passivation, 0.023 g of CdAc_2_ (molar mass: 230.5 g/mol) was added to 1 mL of the previously prepared polar solvent mixture (DMF: DMSO: BTA: 3‐methylpyridine = 35:25:37:3, v/v) and shaken thoroughly until fully dissolved. Ligand‐exchanged PbS CQDs were weighed and redispersed in the Cd‐containing polar solvent to form a concentrated ink at 150 mg/mL. The resulting CQD ink was deposited onto the substrate via spin‐coating at 2500 rpm for 40 s with an acceleration of 1000 rpm s^−1^. After deposition, the films were annealed at 90°C for 15 min to remove residual solvents and enhance film uniformity.

### 4.3 Preparation of Randomly Oriented CNT Film

Arc‐discharged CNT powder (2 mg/mL, Carbon Solution, Inc.) and the conjugated polymer poly[9‐(1‐octylonoyl)‐9H‐carbazole‐2,7‐diyl] (PCz, 2 mg/mL) were co‐dispersed in 500 mL of toluene and sonicated for 30 min. The dispersion was centrifuged at 50 000 *g* for 2 h (Sorvall LYNX6000, Thermo) to remove large aggregates. The supernatant was purified via dynamic liquid‐phase filtration and rinsed with tetrahydrofuran (THF) to eliminate residual impurities. Purified CNTs were redispersed in 1,1,2‐trichloroethane and briefly sonicated again (5 min, 600 W), followed by a second centrifugation to obtain semiconducting CNTs with >99.99 % purity. The CNT solution was then diluted into toluene, and the local‐gate structured substrates were immersed for 48 h. Afterward, the substrates were purged with high‐purity nitrogen (99.999 %), followed by the yttrium oxide coating and decoating (YOCD) process to remove the residual polymer [49]. The resulting randomly oriented CNT film structure is presented in Figure 3A.

### 4.4 Fabrication of CQD Photodiodes and Wafer‐Scale HGFETs

CQD photodiodes were fabricated on ITO‐coated glass substrates to evaluate the p‐i‐n heterojunction characteristics. The substrates were first cleaned via ultrasonic treatment. A ZnO ETL was then deposited onto the ITO surface by magnetron sputtering at 100 W for 30 min. Passivated PbS CQDs were then spin‐coated onto the ZnO layer to form a thin film with a thickness of 100∼150 nm. Subsequently, a PbS‐EDT CQD layer (with an absorption peak ∼880 nm) was deposited atop the PbS‐IBr or PbS‐IBrCd CQD layer, serving as HTL. Finally, an Au back electrode was thermally evaporated and patterned using a shadow mask to define the top electrode area.

Wafer‐scale CNT HGFETs were fabricated on a 4‐inch Si/SiO_2_ wafer using standard photolithography and lift‐off processes. First, local gate electrodes were defined by photolithography, followed by electron beam evaporation (EBE) of a 1/25 nm Ti/Pd stack and subsequent lift‐off. A 10 nm‐thick HfO_2_ gate dielectric was then deposited via atomic layer deposition (ALD). Contact vias were opened through photolithography and reactive ion etching (RIE). A wafer‐scale CNT thin film was deposited as previously described, and device channels were patterned by selective CNT removal using RIE. Source and drain electrodes were formed via photolithography, EBE deposition of 30/40 nm Pd/Au, and a lift‐off process. A high‐κ Y_2_O_3_ layer (6 nm) was prepared by depositing a 3 nm Y film followed by thermal oxidation at 270°C in air for 30 min. The p‐i‐n heterojunction stack was then deposited on top of the Y_2_O_3_ layer using magnetron sputtering and spin‐coating, as described above, as previously described. This included: (1) Sputtering and patterning a 40 nm n‐type ZnO layer; (2) Spin‐coating a 100∼150 nm passivated PbS CQD absorber layer; (3) Spin‐coating a 30 nm p‐type PbS‐EDT CQD layer.

### 4.5 Characterization and Measurement

XPS measurements were performed on an AXIS‐ULTRA DLD‐600 W Ultra spectrometer (Kratos Co., Japan). Optical absorption spectra of PbS CQDs were recorded with a SolidSpec‐3700 UV–vis–NIR spectrophotometer, while PL spectra were measured using a QuantaMaster 8000 steady‐state/transient modular fluorescence spectrometer. TEM and SEM were conducted using a Talos F200X (Thermo Scientific, 300 kV) and a ZEISS Sigma 300, respectively. CNT density was analyzed usingh NMMeasure software. DLCP measurements were carried out at room temperature with a Keithley 4200 semiconductor analyzer, using a 10 kHz frequency. The d.c. bias ranged from −0.4 to 0.4 V (or 0.8 V), and the a.c. bias amplitude was set between 20 and 40 mV. Photocurrent spectroscopy was performed using an SR830 lock‐in amplifier in conjunction with an SR540 chopper and a tungsten lamp equipped with a monochromator, all controlled via LabVIEW. *J*‐*V* characteristics of the photodiodes were measured using an Agilent B1500A semiconductor characterization system. Optoelectronic measurements employed a supercontinuum laser source (NKT, 400–2100 nm), combined with an IHR320 monochromator and a Thorlabs NIR power attenuator (Ø25 mm). Beam profiles were captured using an InGaAs camera (First Light Vision C‐Red3, Figure S10 and Table S2) and independently verified by the knife‐edge method (see Figure S10). Electrical measurements of HGFETs were performed under ambient conditions using a Keithley 4200 analyzer. Noise spectra were obtained and cross‐validated using PDA FS380 and Keysight E4727B integrated systems. Response times and −3 dB bandwidths were determined with an Agilent DSO7054A oscilloscope.

### 4.6 Calculation and Benchmarking of D*

D* quantifies the signal‐to‐noise ratio of a photodetector normalized by unit incident optical power, unit area, and unit bandwidth. It is widely considered the most important figure of merit for evaluating photodetector performance. For point‐detection applications, D* is typically calculated using the relation: $D^{*}=R\sqrt{A\Delta f}/I_{n}$, where Δ*f* is the noise‐equivalent bandwidth (commonly set to 1 Hz), and *I_n_* is the noise current spectral density (A Hz^−1/2^) measured at a given frequency. However, in practical photodetectors, especially nanoscale devices, low‐frequency noise is often dominated by 1/*f* noise, which changes markedly below 1 kHz. This makes the calculated D* highly dependent on the chosen measurement frequency, potentially leading to inconsistency or overestimation. In this work, the photodiodes and HGFETs under study exhibit different bandwidths depending on their structure and biasing conditions. To avoid overestimation and better reflect the intrinsic sensitivity of each detector, we adopt a frequency‐integrated D* model commonly used in imaging applications:$\;D^{*}=R\sqrt{Af_{B}}/\sqrt{\int_{0}^{f_{B}}I_{n}^{2}\left(f\right)df}$ [40]. Here, *f_B_* denotes the −3 dB cutoff frequency, which defines the effective detection bandwidth. For HGFETs, *f_B_* was measured directly, while for the photodiodes in Figure 2, *f_B_* was estimated from the response time (*τ* ≈ *t*_rise_) in Figure S8b using the empirical relation *f_B_* = 0.35/*τ*, as the available chopped light source is limited to 30 kHz. This formulation accounts for the total noise power within the device's operational range, yielding a more accurate and comparable measure of detectivity across different device architectures.

## Conflicts of Interest

The authors declare no conflicts of interest.

## Supporting information




**Supporting File**: advs73828‐sup‐0001‐SuppMat.docx.

## Data Availability

The data that support the findings of this study are available from the corresponding author upon reasonable request.
