# Supplementary material for: Highly Sensitive Heterojunction‐Gated Phototransistor With Detection Wavelength Ranged From 350 to 1700 Nm
Source: Adv Sci (Weinh). 2026 Jan 12;13(17):e22627. doi: 10.1002/advs.202522627 (PMC13042453; doi:10.1002/advs.202522627)
Supplement: Supplementary file 1 — Supporting File: advs73828‐sup‐0001‐SuppMat.docx. [file ADVS-13-e22627-s001.docx]

**Supplementary Materials for**

**Highly Sensitive Heterojunction-Gated Phototransistor with Detection Wavelength Ranged from** **350 to 1700 nm**

Hongkun Duan^1,2,#^, Wenyu Zhang^3,#^, Tao Luo^4^, XiaoLu Xia^2^, Qianxi Yang^4^，Ying Yan^1,2^, Shengmei Gao^4^,Xitian Yin^2^, Yixiao Niu^3^, Zhiyu Zhao^3^, Jianbing Zhang^3,^*, Haobin He^5^, Jiang Tang^6^, Ying Wang^2,5,^*, and Zhiyong Zhang^4,5,^*

^1^School of Integrated Circuits, Beijing University of Posts and Telecommunications, Beijing, China.

^2^Key Laboratory of Luminescence & Optical Information, Ministry of Education, School of Physical Science and Engineering, Beijing Jiaotong University, Beijing, China.

^3^School of Integrated Circuits, Huazhong University of Science and Technology, Hubei, China.

^4^Key Laboratory for the Physics and Chemistry of Nanodevices and Center for Carbon-based Electronics, School of Electronics, Peking University, Beijing, China.

^5^Chongqing Institute of Carbon-Based Integrated Circuits Peking University, Chongqing, China.

^6^Wuhan National Laboratory for Optoelectronics (WNLO), Huazhong University of Science and Technology, Wuhan, China.

e-mail: [jbzhang@hust.edu.cn](mailto:jbzhang@hust.edu.cn), [yingw@bjtu.edu.cn](mailto:yingw@bjtu.edu.cn), and [zyzhang@pku.edu.cn](mailto:zyzhang@pku.edu.cn)


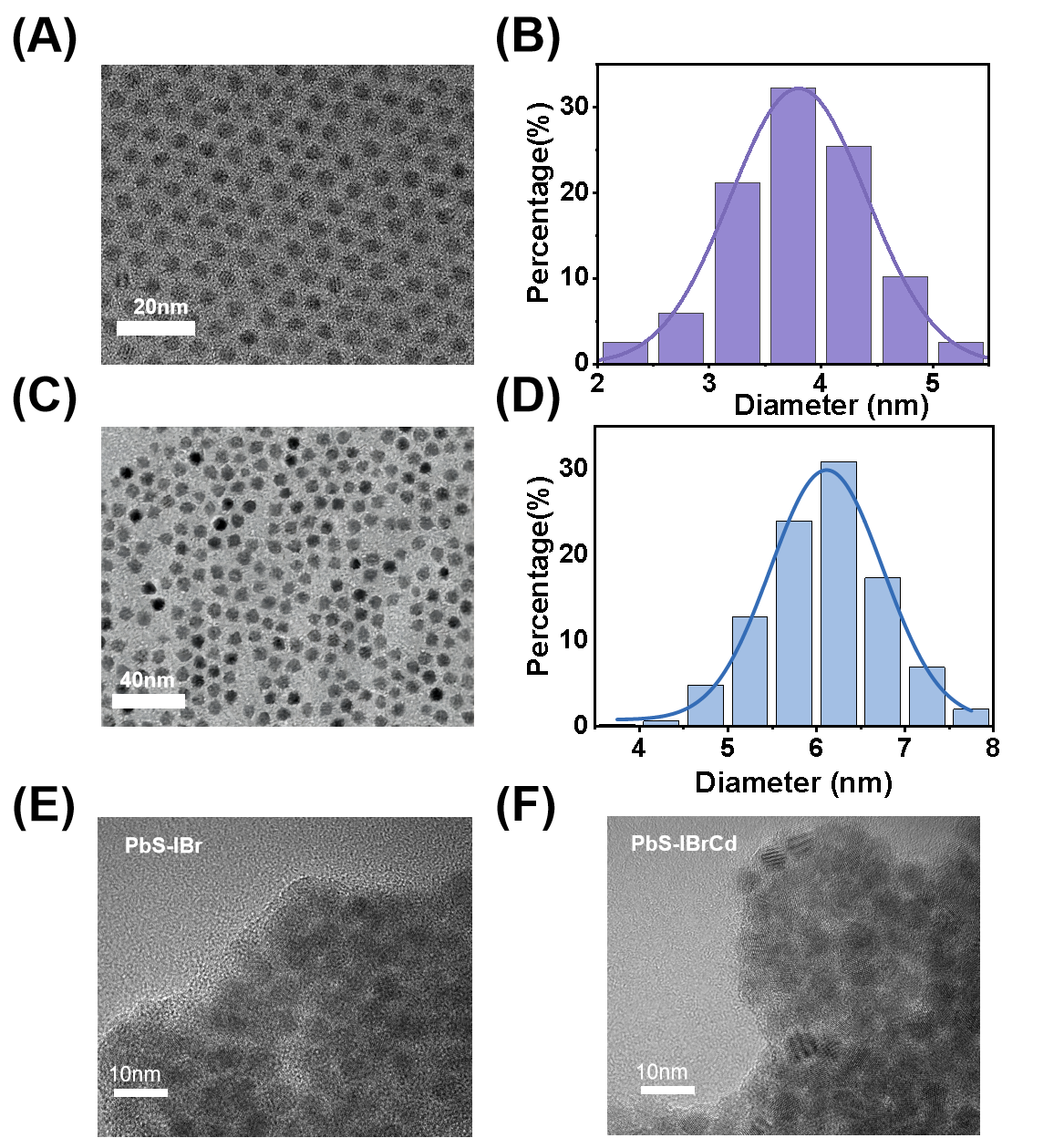


**Figure S1. Morphology and particle size distribution of PbS CQDs**. (A, C) Transmission electron microscope (TEM) images of PbS CQDs with excitonic absorption peaks at 1300 nm (A) and 1650 nm (C). (B, D) Corresponding size distribution histograms, showing average diameters of 3.8 nm (B) and 6.0 nm (D), respectively. (E, F) TEM images of PbS–IBr (E) and PbS–IBrCd CQDs (F) with absorption peaks at 1650 nm.


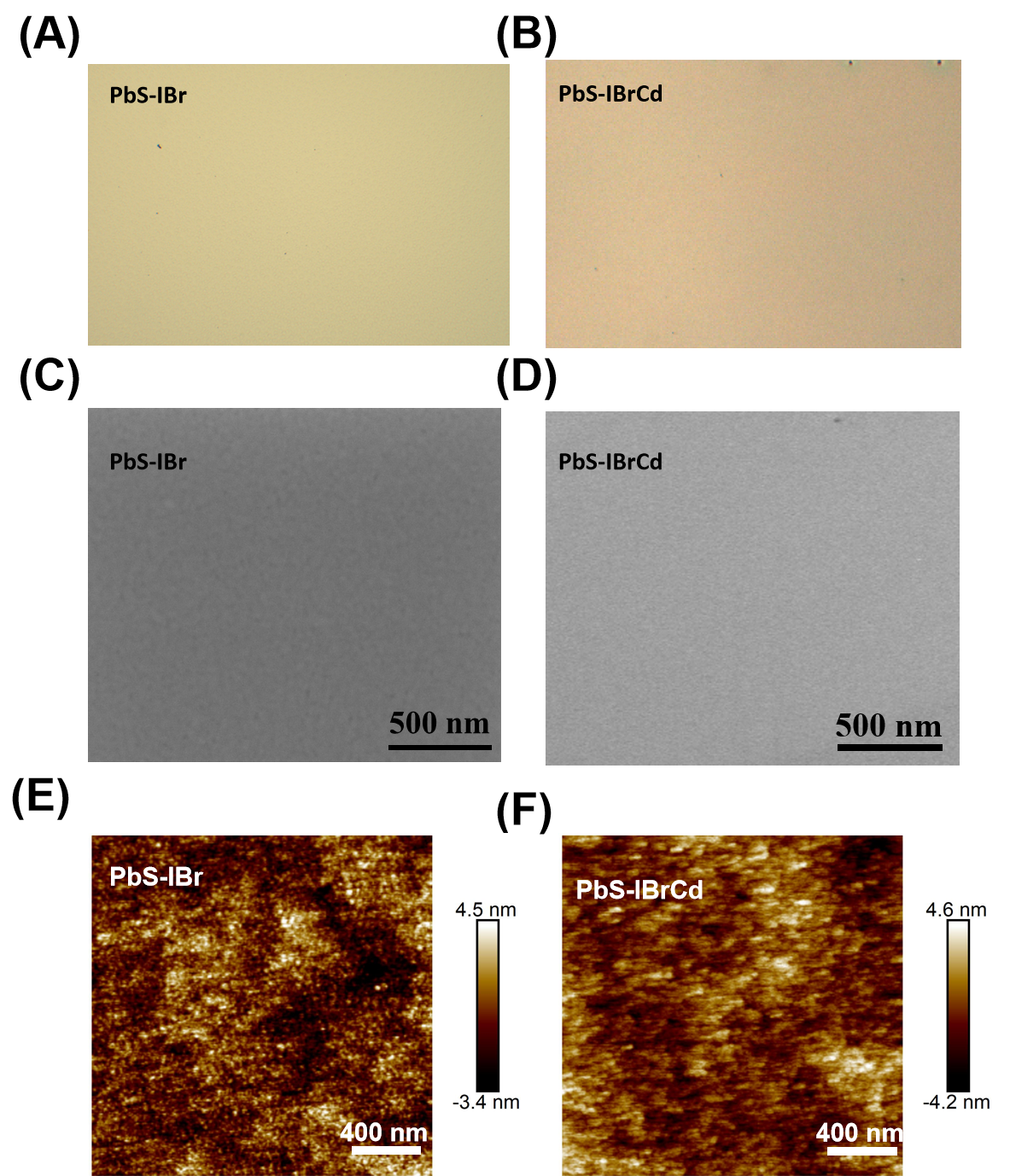


**Figure S2. Optical, SEM and AFM images of PbS–IBr (A, C, E) and PbS–IBrCd CQD films (B, D, F).**


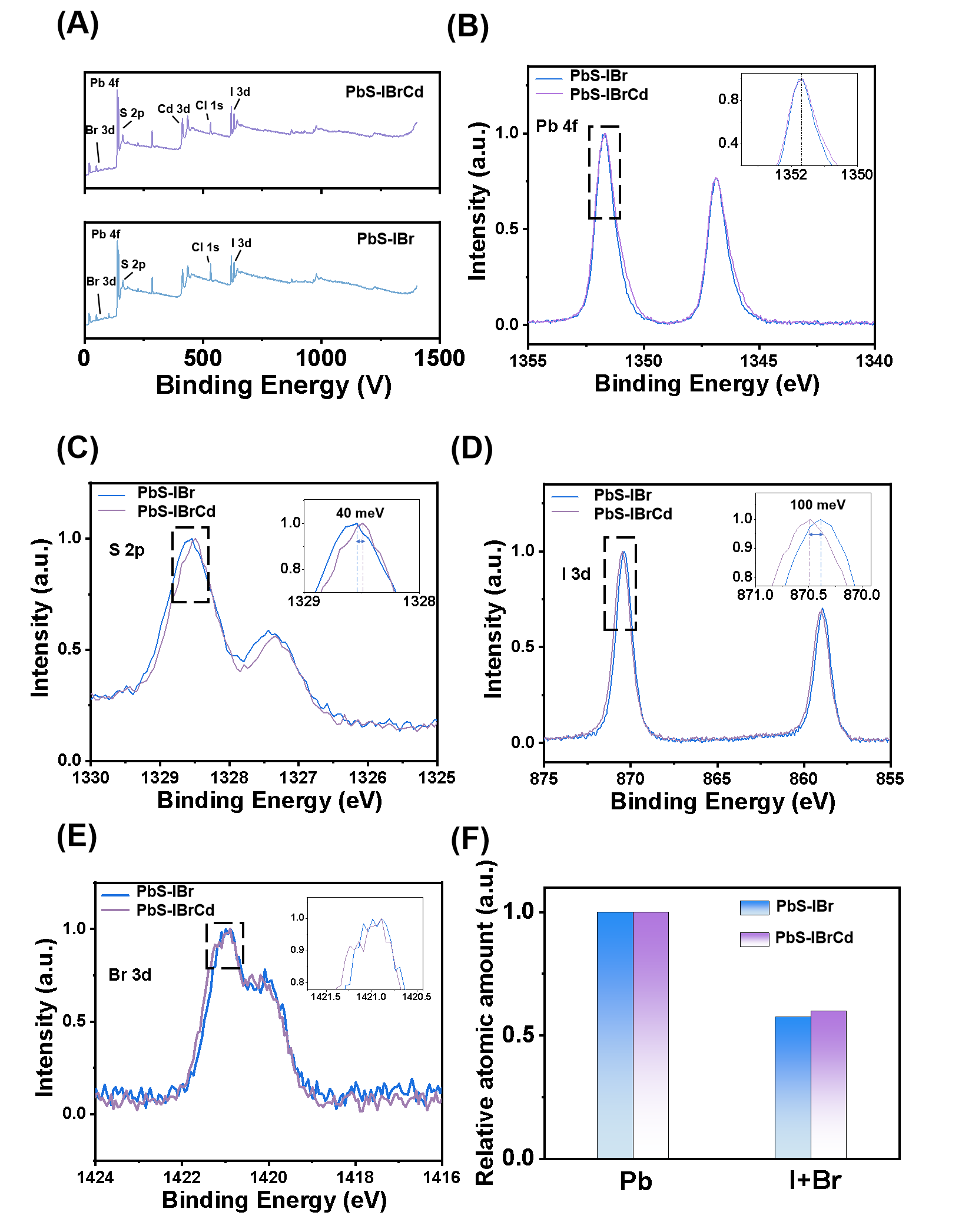


**Figure S3. XPS analysis of PbS CQD films before and after CdAc_2_ treatment.** (A) Wide-scan XPS spectra. High-resolution XPS spectra showing the Pb 4f (B), S 2p (C), I 3d (D), and Br 3d (E). No discernible binding-energy shift is observed for the Pb 4f core level upon Cd introduction. The I 3d peak shifts toward higher binding energy by ~100 meV, while the S 2p peak shifts toward lower binding energy by ~40 meV. The Br 3d signal is relatively weak, making clear peak shifts difficult to discern. (F) Atomic ratios of Pb to halides (I^-^ and Br^-^), showing changes in surface composition induced by CdAc_2_ treatment.


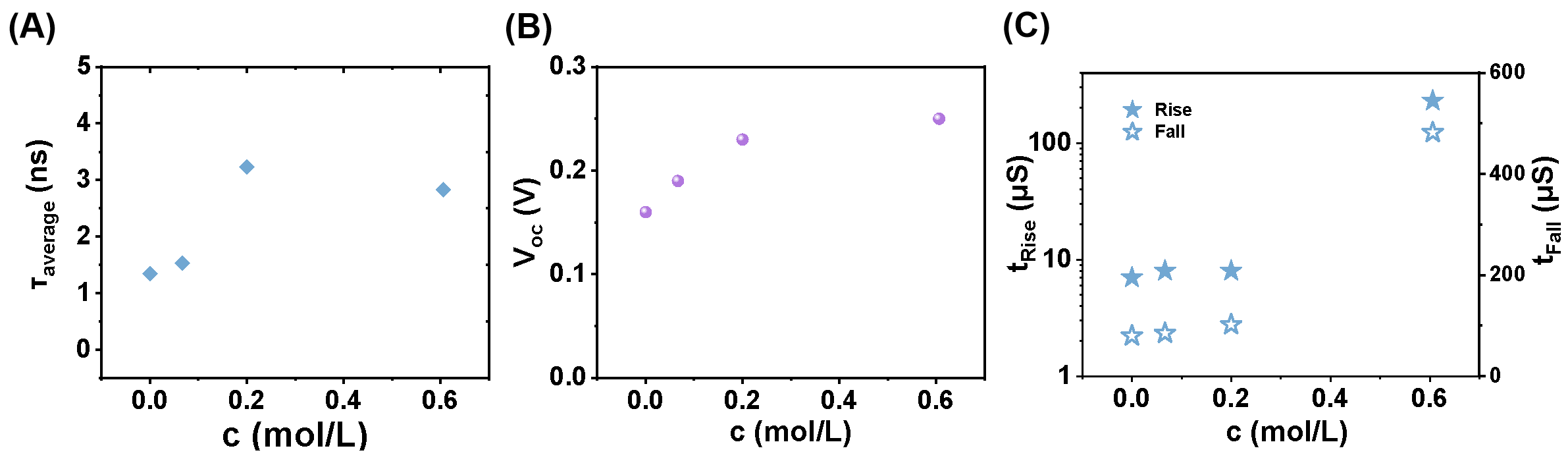


**Figure S4. Optoelectronic characterization of** **PbS-IBrCd CQD films with varying Cd additive concentrations.** (A) Carrier lifetimes extracted from TRPL measurements. (B) Open-circuit photovoltages of PbS-IBrCd photodiodes under 1650 nm illumination at an intensity of 340 μW cm^-2^. (C) Temporal response characteristics (rise and fall times) measured at a bias of –0.1 V.

**Table S1. Extracting average carrier lifetimes from TRPL measurements.**

| Sample | 𝜏_1_ (ns) | R_A1_ (%) | 𝜏_2_ (ns) | R_A2_ (%) | 𝜏_avg_ (ns) |
| --- | --- | --- | --- | --- | --- |
| PbS-CdIBr | 0.947 | 25.8% | 5.041 | 74.2% | 3.9 |
| Control | 0.497 | 31.9% | 2.138 | 68.1% | 1.6 |

In Figure 1C and 1D, the PL decay curves were well fitted by a biexponential function: *y*=*A*_1_*e*^-^*^t^*^/^*^τ^*^1^ + *A*_2_*e*^-^*^t/τ^*^2^, where *A*_1_ and *A*_2_ are the amplitudes, and *τ*_1_ and *τ*_2_ represent the fast and slow decay lifetimes. The average carrier lifetime was calculated using: *τ*_avg_=∑*A*_i_*τ*_i_^2^/∑*A*_i_*τ*_i_ (*i*=1,2). The relative contribution of each decay component was obtained from *R*_A1_=*A*_i_*τ*_i_/∑*A*_i_*τ*_i_ (*i*=1,2). Here, the fast decay component (*R*_A1_) is primarily attributed to nonradiative recombination, whereas the slow decay component (*R*_A2_) is associated with interdot charge transfer and intrinsic radiative recombination.


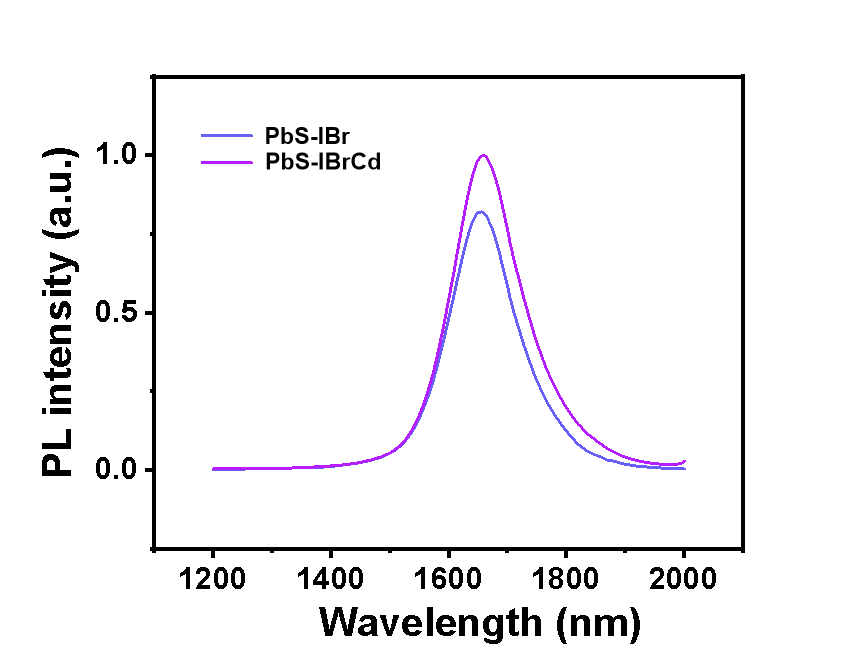


**Figure S5**. Steady-state PL spectra of the PbS-IBr and PbS-IBrCd films.


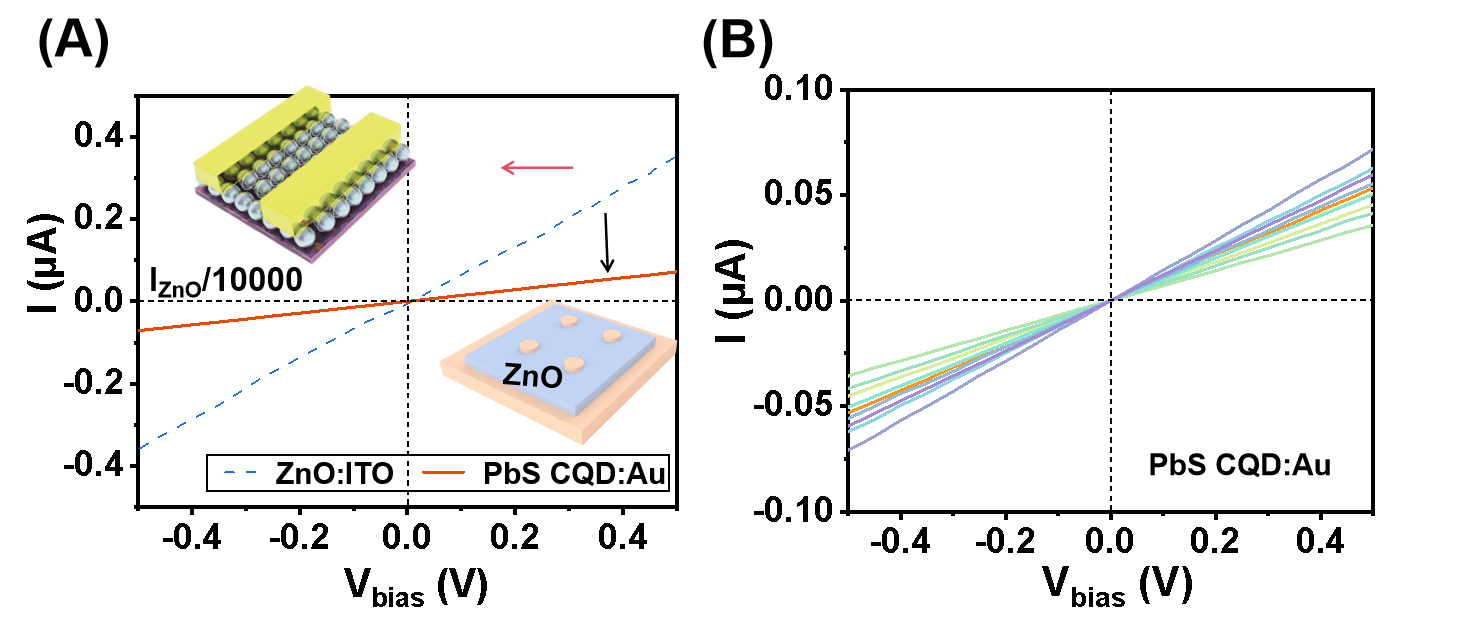


**Figure S6. Current-voltage characteristics under low bias, demonstrating contact behavior in the photodiodes.** (A) Ohmic contacts are confirmed between ZnO and ITO (dashed line), as well as between Au and the EDT-treated PbS CQD layer (solid line). Inset: Cross-sectional schematic of the device architecture. (B) Current-voltage characteristics of other 10 devices, showing ohmic contact between Au and PbS-EDT film.


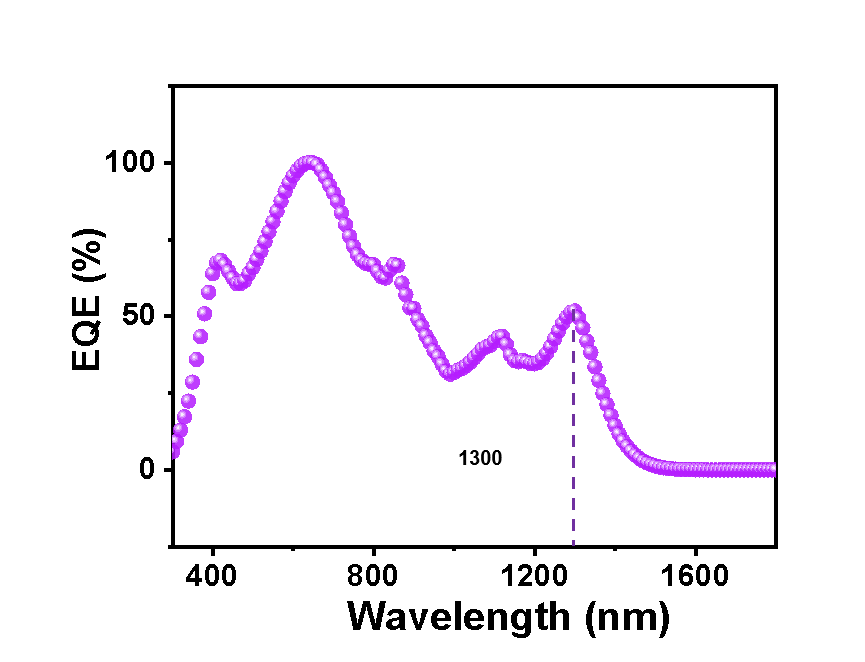


**Figure S7.** EQE spectra for a 3.8 nm PbS-IBr control CQD photodiode measured at -0.1 V bias.


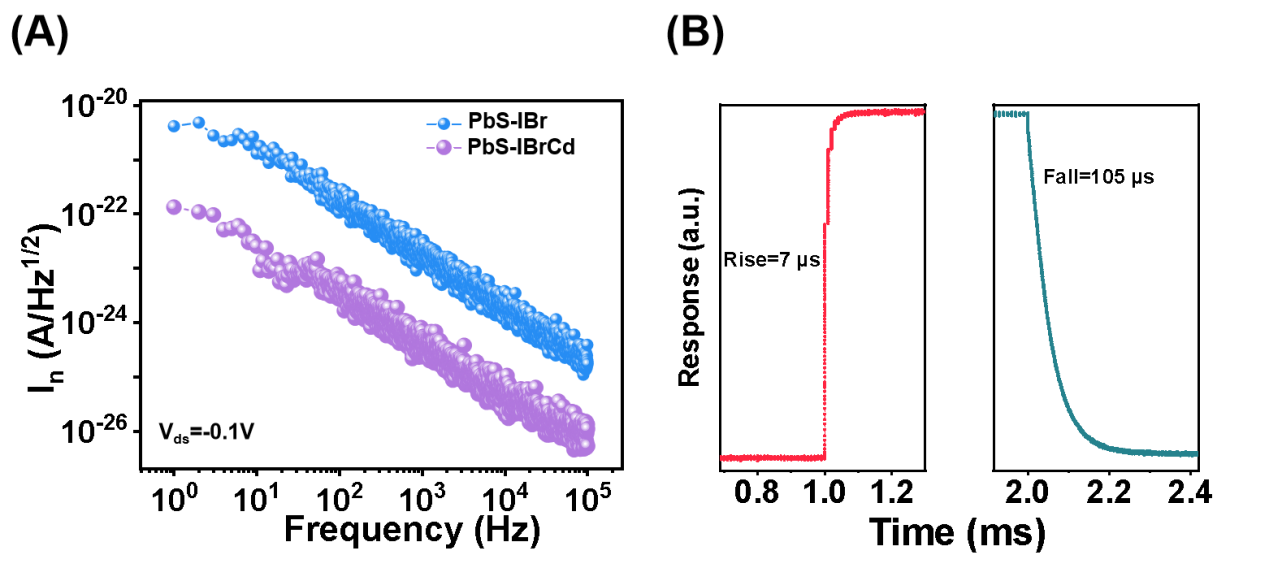


**Figure S8. Noise and temporal response characteristics of PbS CQD photodiodes.** (A) Noise current spectra of PbS-IBr (dashed line) and PbS-IBrCd (solid line) devices measured at $V_{ds}$= -0.1 V. (B) Normalized photocurrent response of the optimized device, showing a rise time of 7 µs and a fall time of 105 µs at $V_{ds}$= -0.1 V.

**Fitting Photocurrent Using a Combined PN Junction and FET Model**

The relationship between the open-circuit voltage ($V_{oc}$) of the p-i-n heterojunction and incident light intensity can be calculated using the expression:

$V_{oc}=\frac{k_{B}T}{q}ln\left( \frac{\eta qAP_{light}}{h\nu I_{0}}+1 \right)$ (1)

where $k_{B}$ is the Boltzmann constant, $T$ is the temperature, $A$ is the optical area, $I_{0}$ is the reverse saturation current, $\eta$is the external quantum efficiency, $P_{light}$is the incident power density, $q$ is the elementary charge, and $h\text{ν}$ is the energy of one photon.

In the subthreshold region of a FET, the relationship between the drain-source current ($I_{ds}$) and photovoltage-induced threshold drift (${\Delta V}_{th}$) can be expressed as:

$logI_{ds}=\frac{{\Delta V}_{th}}{SS}+logI_{ds0}$ (2)

where $SS$ is the subthreshold swing, $I_{ds0}$ is the initial drain-source current or dark current. For an HGFET detector based on the opto-electronic decoupling model, the relationship between ${\Delta V}_{th}$ and $V_{oc}$ can be calculated by:

${\Delta V}_{th}=\alpha V_{oc}$ (3)

where $\alpha$ is the coupling coefficient, representing the effective utilization of photovoltage. The expression for $I_{ds}$ can be derived by substituting expressions (1) and (3) into expression (2):

$I_{ds}=\left[ \left( \frac{\eta qAP_{light}}{h\nu I_{0}}+1 \right)^{\frac{2.3\alpha k_{B}T}{qSS}} \right]I_{ds0}$ (4)

The coefficient 2.3 in the expression is a result of converting the natural logarithm (*ln*) to the base-10 logarithm (*log*). The expression for $I_{ph}$ in the subthreshold region can thus be derived as:

$I_{ph}=\left[ \left( \frac{\eta qAP_{light}}{h\nu I_{0}}+1 \right)^{\frac{2.3\alpha k_{B}T}{qSS}}-1 \right]I_{ds0}=[{(aP_{light}+1)}^{\frac{b}{SS}}-1]I_{ds0}$ (5)

where $a=\eta qA/(h\nu I_{0})$ and $b=2.3\alpha k_{B}T/q$.

In the linear region of the FET, the relationship between the drain-source current and threshold drift is given by:

$I_{ds}=g_{m}{\Delta V}_{th}$+$I_{ds0}$ (6)

where $g_{m}$ is the transconductance of the photovoltage-gated CNTs FET. The expression for $I_{ph}$ can be derived in the linear region as:

$I_{ph}=\frac{g_{m}\alpha k_{B}T}{q}ln\left( \frac{\eta qAP_{light}}{h\nu I_{0}}+1 \right)\sim lnP_{light}$ (7)

In Figure 3D, the photocurrent–light power relationship follows the functional forms described by Equations 5 and 7. The threshold voltage shift induced by photovoltage modulation in the transfer curves of the HGFET under illumination (Figure S9) further supports the proposed model.


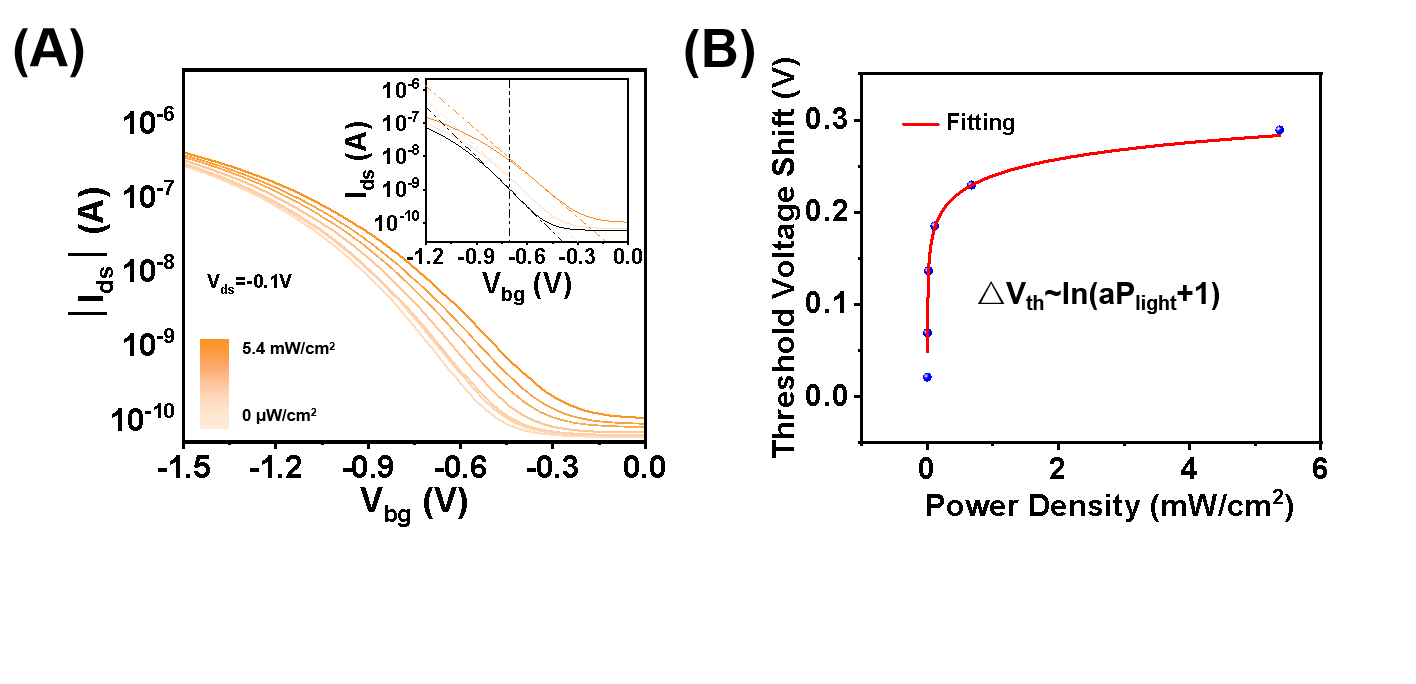


Figure S9. (A) Transfer characteristics of the HGFET measured in the dark and under 1650 nm illumination at increasing incident power densities. (B) Extracted light-induced threshold voltage shift as a function of incident power density.

**Determination of Beam Spot Size**

Accurate determination of the beam spot size is critical for calculating the optical power density and the device responsivity. To avoid underestimating the illuminated area, we standardized the measurement conditions and performed an independent cross-validation in the revised manuscript. First, for the infrared camera measurements, we consistently used the manufacturer-recommended high-gain mode, which captures the main body of the Gaussian beam profile. As shown in Figure S10, a calibrated reference object with a 40 μm length appears as 78 pixels in the infrared camera image, yielding a pixel-to-length conversion factor. Under identical camera settings, the laser spot diameter was measured to be 137 pixels, corresponding to a spot diameter ~70.3 μm. For comparison, measurements performed using the low-gain mode yield a smaller spot diameter of ~56 μm, confirming that gain selection can influence the extracted spot size.

To further ensure the accuracy and objectivity of the spot-size estimation, we performed an independent knife-edge measurement as a cross-check (Figure S10G). A sharp blade mounted on a precision translation stage was positioned at the same plane as the detector to ensure that the measured beam profile corresponded exactly to the illumination conditions during device testing. As the blade was translated laterally across the beam, the transmitted optical power was recorded, yielding a power–position curve. For a Gaussian beam, the beam diameter is related to the measured clip width D_c_, defined as the distance between the positions at which the transmitted power increases from 10% *P*_0_ to 90% *P*_0_, where *P*_0_ is the total beam power. Under these conditions, the full beam diameter *D* is given by *D*=1.561*D*_c_.^[13]^ Using this method, we obtained a clip width *D*_c_ of 46 μm and a beam diameter of 71.8 μm. The close agreement between the knife-edge result (~71.8 μm) and the high-gain camera-based measurement (~70 μm) confirms that the adopted spot size is reasonable. Therefore, we use a conservative spot diameter of ~70 μm for responsivity and detectivity calculations.

**Table S****2. Calibration of incident light intensity (1650 nm).**

Note: Power density was controlled using aperture slots (Slots 1–7) and neutral density filters (NENIR30B/NENIR60B; Thorlabs), and calibrated via Ge photodetector (PM100D) with InGaAs reference diode (FGA015). The symbol "–" denotes unmeasurable signals or excluded configurations.


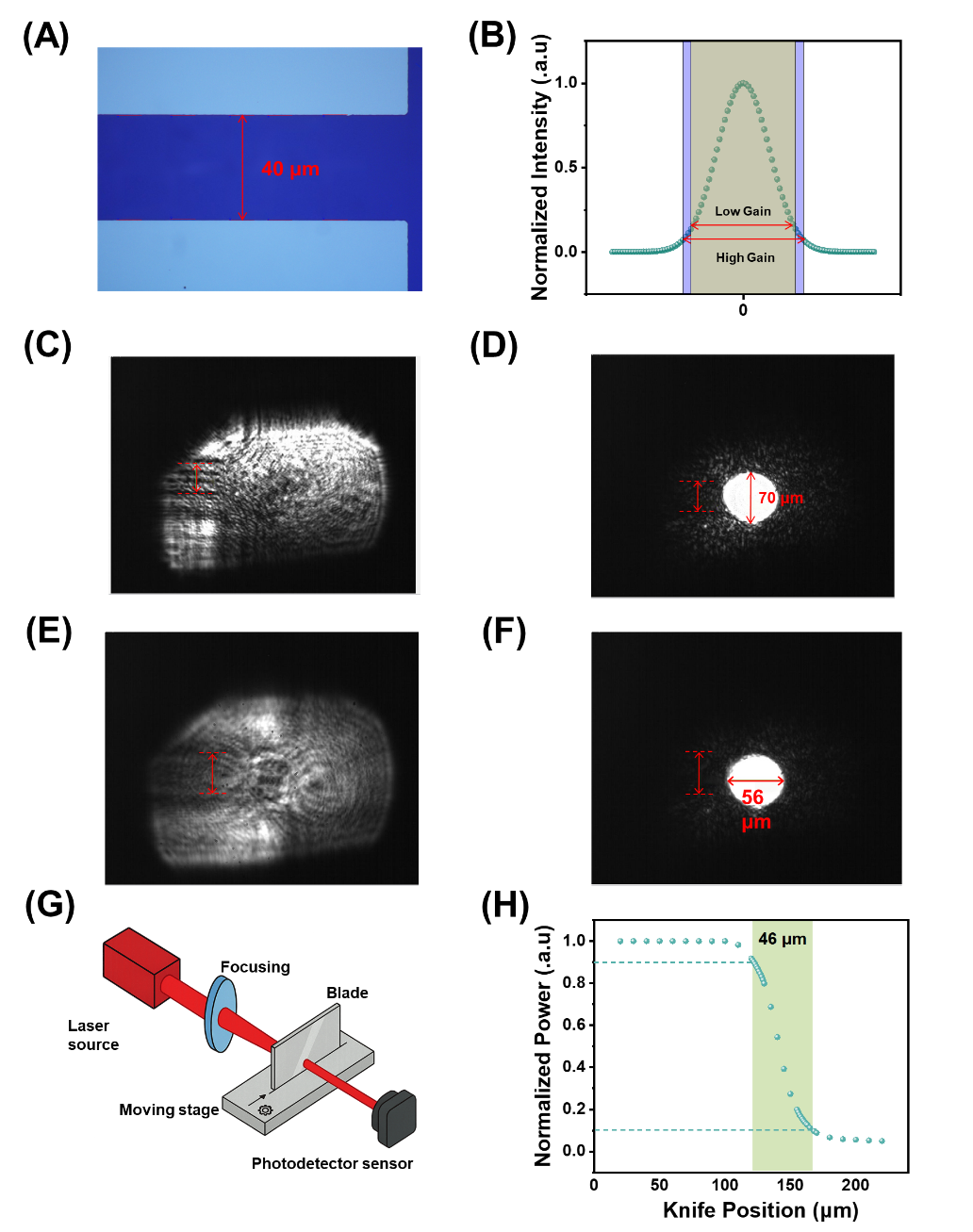


**Figure S10.** Calibration and verification of the laser spot size. (A) Optical microscope image of the reference object used for calibration. (B) Schematic illustration showing the dependence of the spot size on camera gain with a Gaussian intensity distribution. (C) Infrared camera image of the reference object acquired in the high-gain mode. (D) Focused laser spot image acquired in the high-gain mode. (E) Infrared camera image of the reference object acquired in the low-gain mode. (F) Focused laser spot image acquired in the low-gain mode. (G) Schematic of the knife-edge measurement used for beam spot verification.^(1)^ (H) Normalized optical power as a function of knife-edge displacement.


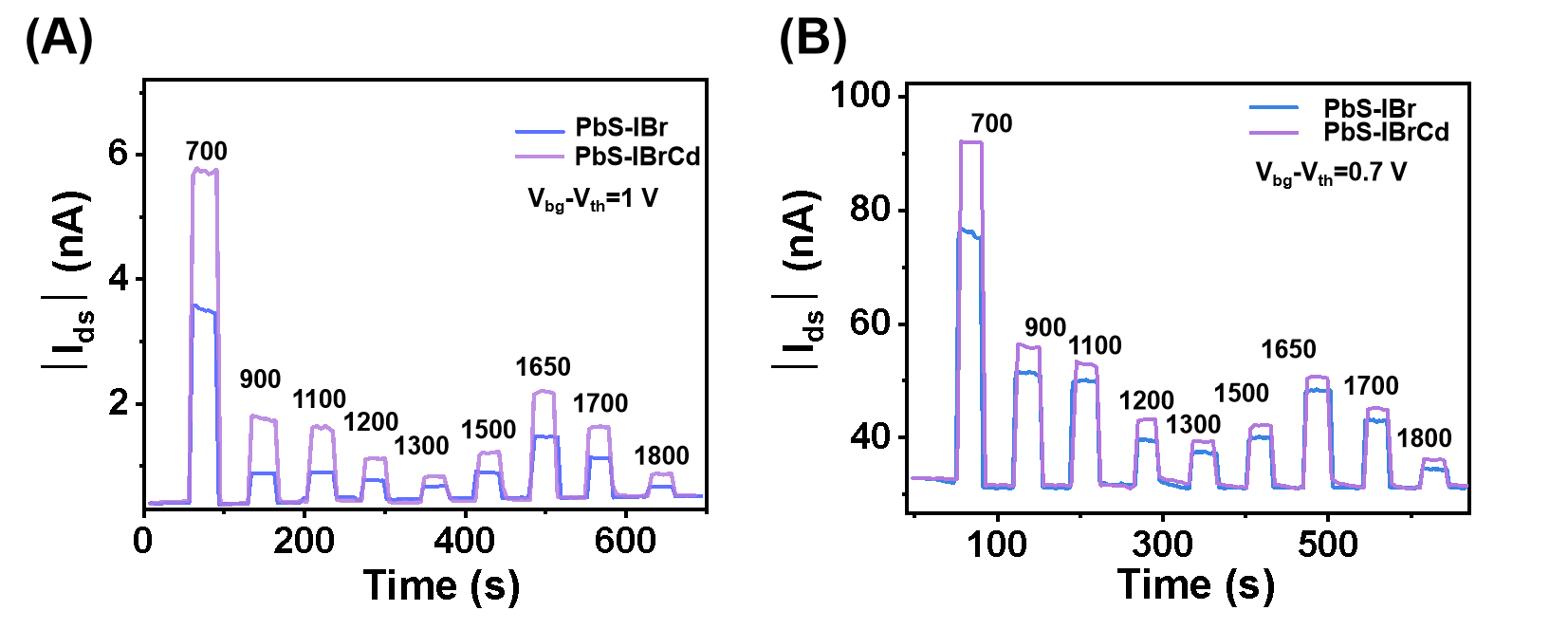


**Figure S11. Time-resolved photoresponse of PbS-IBrCd and PbS-IBr HGFETs under broadband illumination from 700 to 1800 nm.** Photoresponse measured at $V_{bg}-V_{th}$ = 1.0 V (A) and$V_{bg}-V_{th}$ = 0.7 V (B).


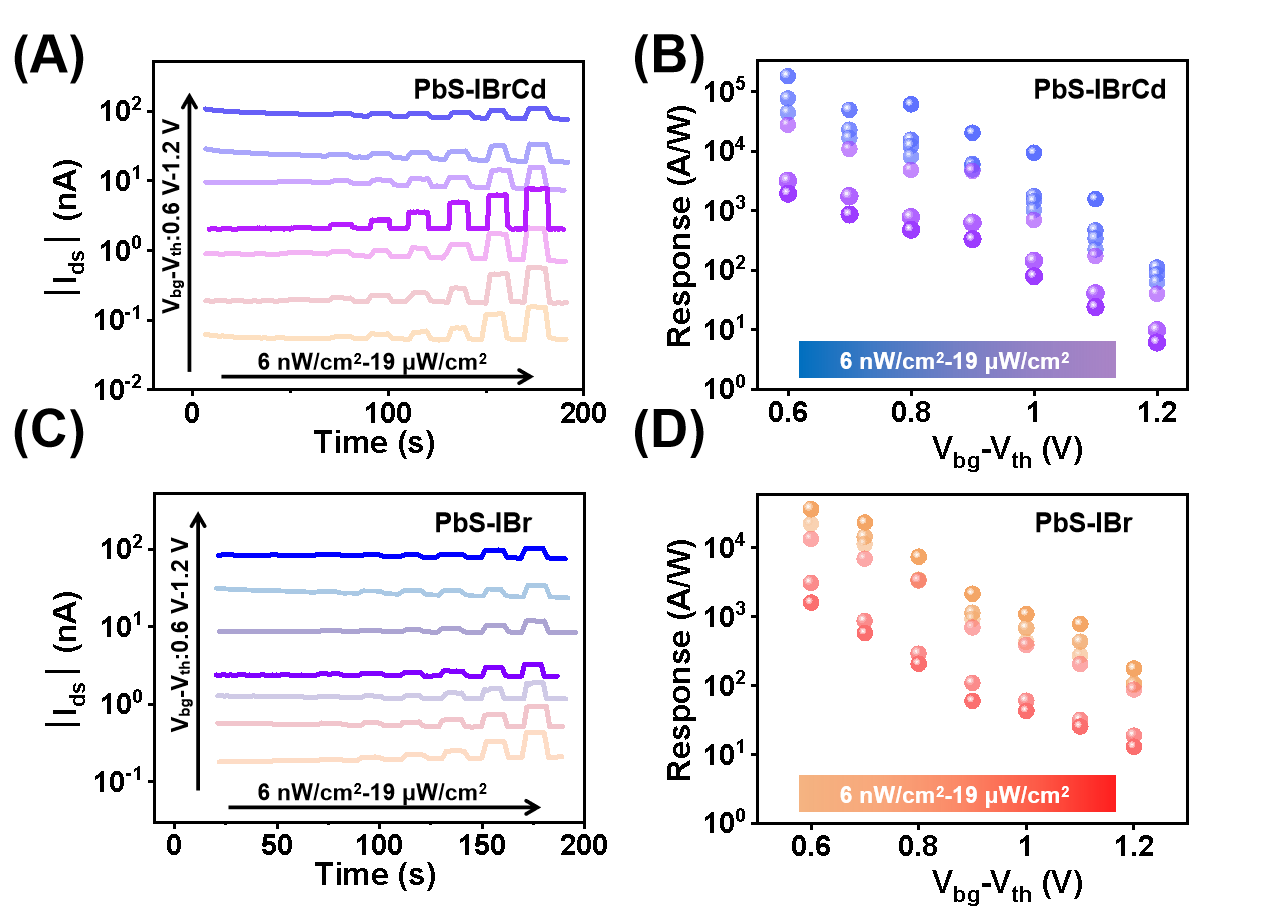


**Figure S12. Time-resolved photocurrent response of HGFET devices under low-intensity illumination (6.4 nW cm^-2^ to 19 μW cm^-2^) across**$\boldsymbol{V}_{\boldsymbol{bg}}\boldsymbol{-}\boldsymbol{V}_{\boldsymbol{th}}$**of 0.6-1.2 V.** Transient photocurrent responses of PbS-IBrCd (A) PbS-IBr (C) devices. Corresponding responsivity spectra (B and D) extracted from (A) and (C), respectively.


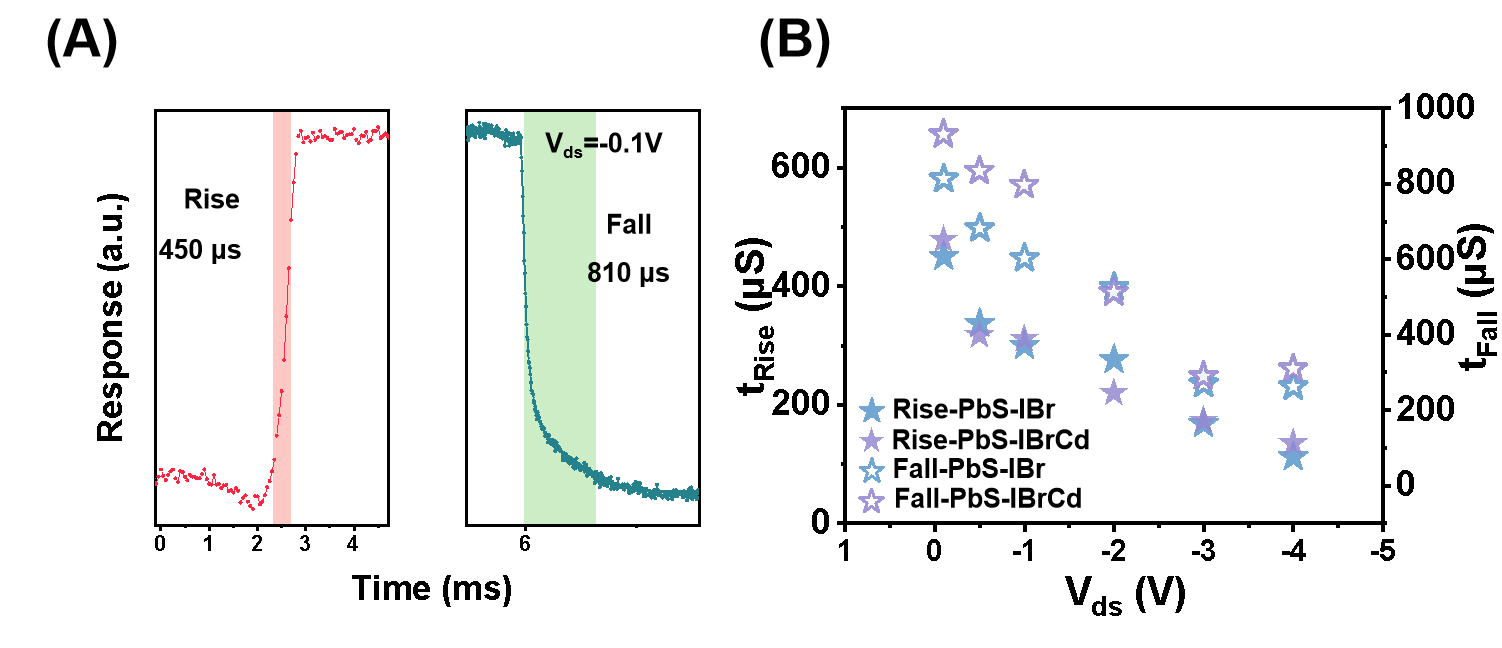


**Figure S13. Photocurrent response dynamics of the HGFET devices.** (A) Normalized photocurrent response showing a rise time of 450 µs and fall time of 810 µs at $V_{ds}$= -0.1 V. (B) Dependence of response time on the source-drain voltage.


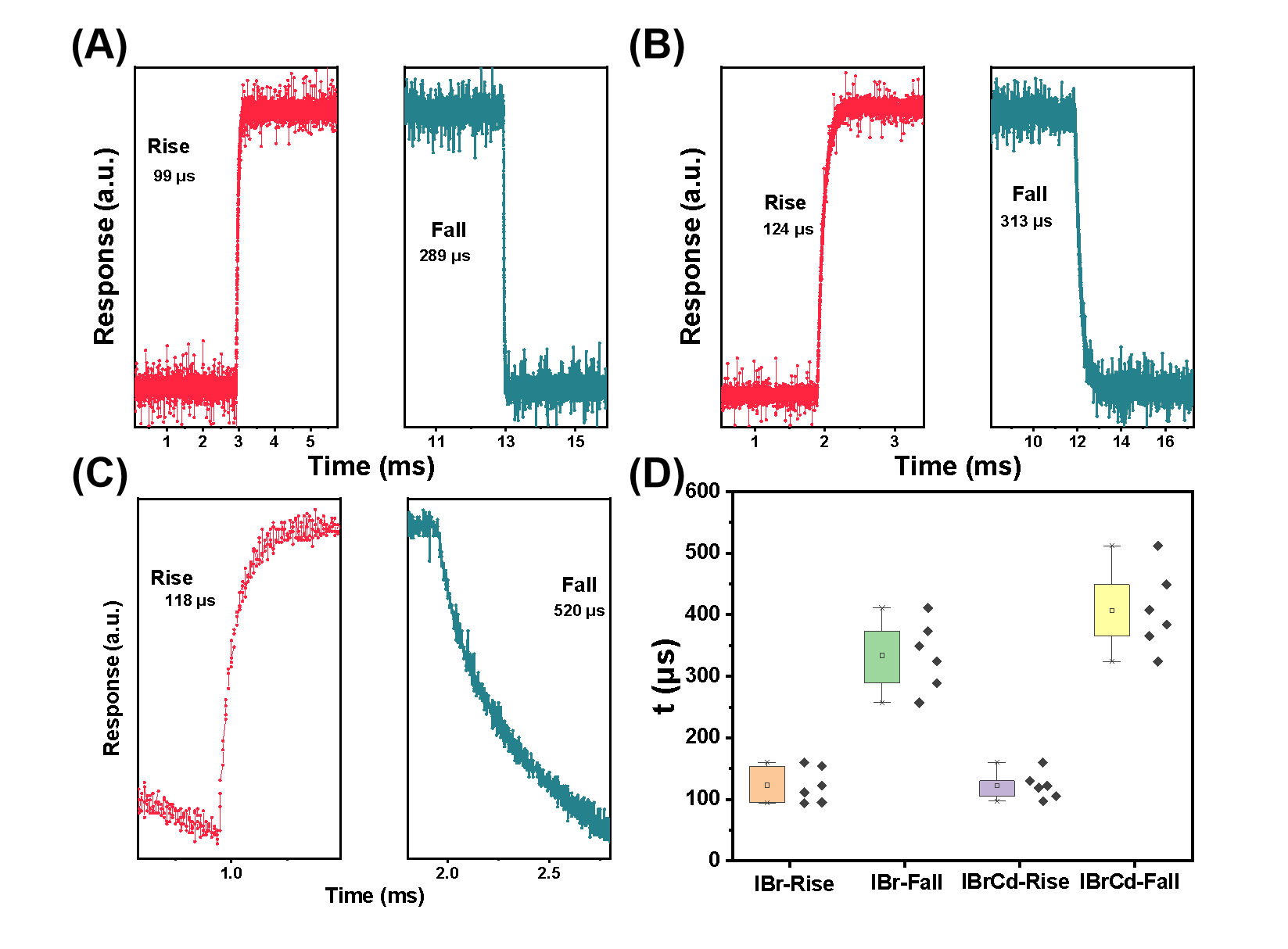


**Figure S14.** Transient photoresponse of PbS CQD photodiodes measured under zero bias. (A, B) Transient photoresponse of the PbS-IBr and PbS-IBrCd CQD photodiodes, respectively. (C) Transient photoresponse of another PbS-IBrCd device. (D) Rise and fall times of multiple photodiodes.


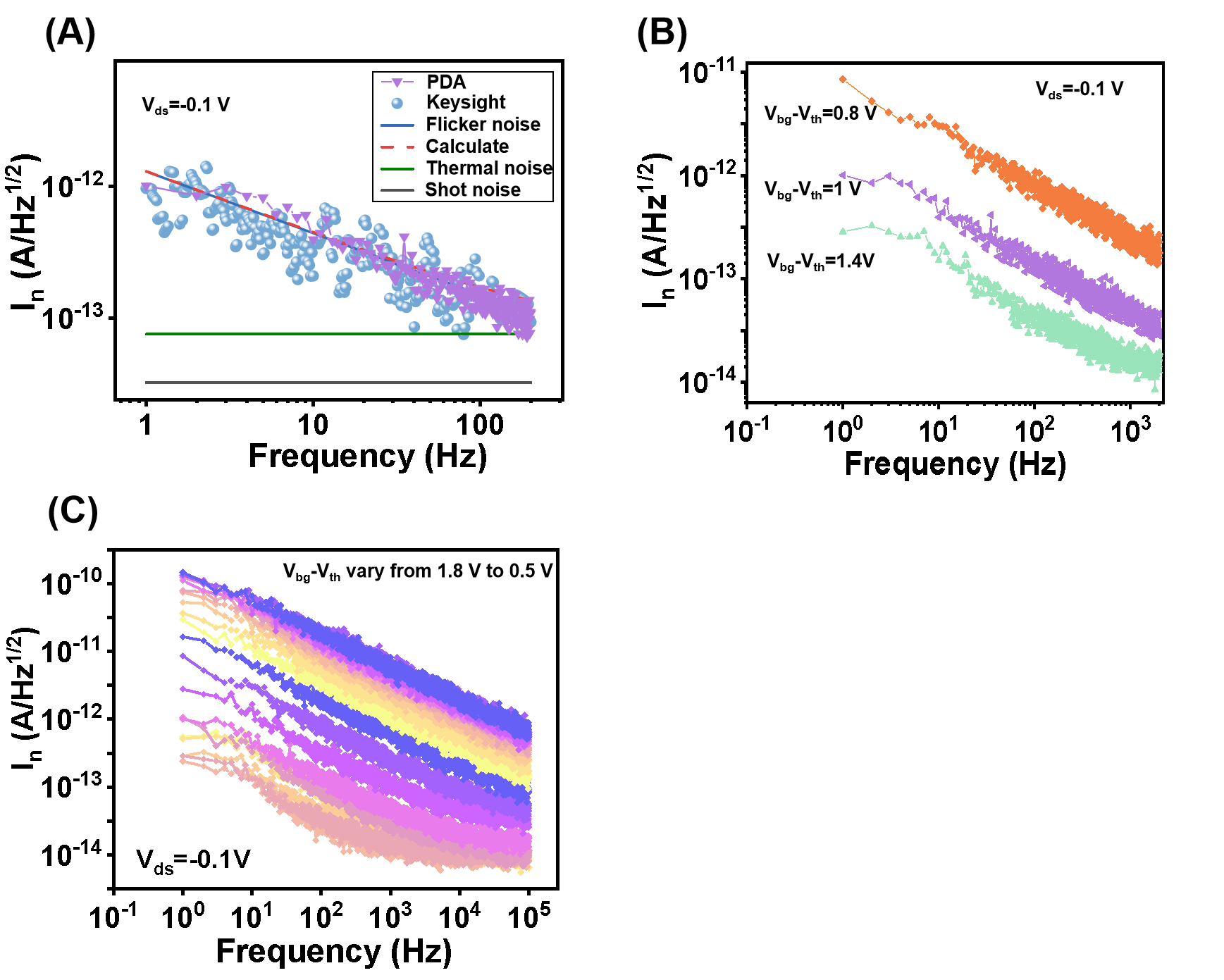


**Figure S15. Noise characteristics of HGFET devices measured at** $\boldsymbol{V}_{\boldsymbol{ds}}$**= -0.1 V.** (A) Frequency-dependent noise spectrum at $V_{bg}-V_{th}$ = 1.0 V, showing theoretical shot noise, flicker noise, and thermal noise limits. Current noise density spectra measured at three representative$V_{bg}-V_{th}$values (B) and at varying$V_{bg}-V_{th}$values from 1.8 V to 0.5 V (C).


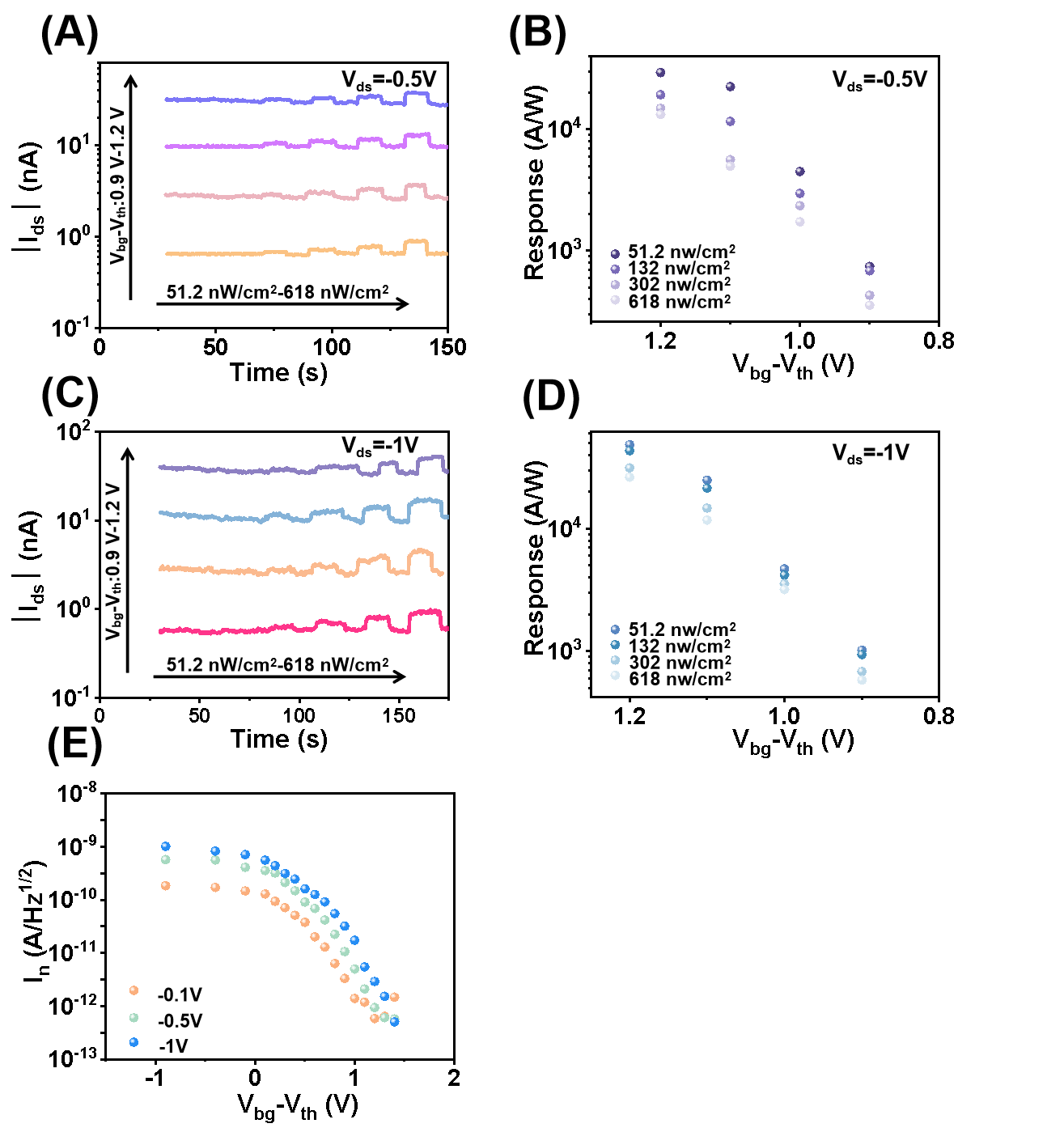


**Figure S16. Time-resolved photocurrent response and noise characteristics of the HGFET under low-intensity illumination (51.2 nW cm^-2^ to 618 nW cm^-2^) across**$\boldsymbol{V}_{\boldsymbol{bg}}\boldsymbol{-}\boldsymbol{V}_{\boldsymbol{th}}$**values from 0.9 V to 1.2 V.** Transient responses measured at $V_{ds}$= -0.5 V (A) and $V_{ds}$= -1.0 V (C). (B, D) Corresponding responsivity spectra extracted from (A) and (C), respectively. (E) Measured current noise spectra of the HGFET device at varying back-gate and drain-source voltages.


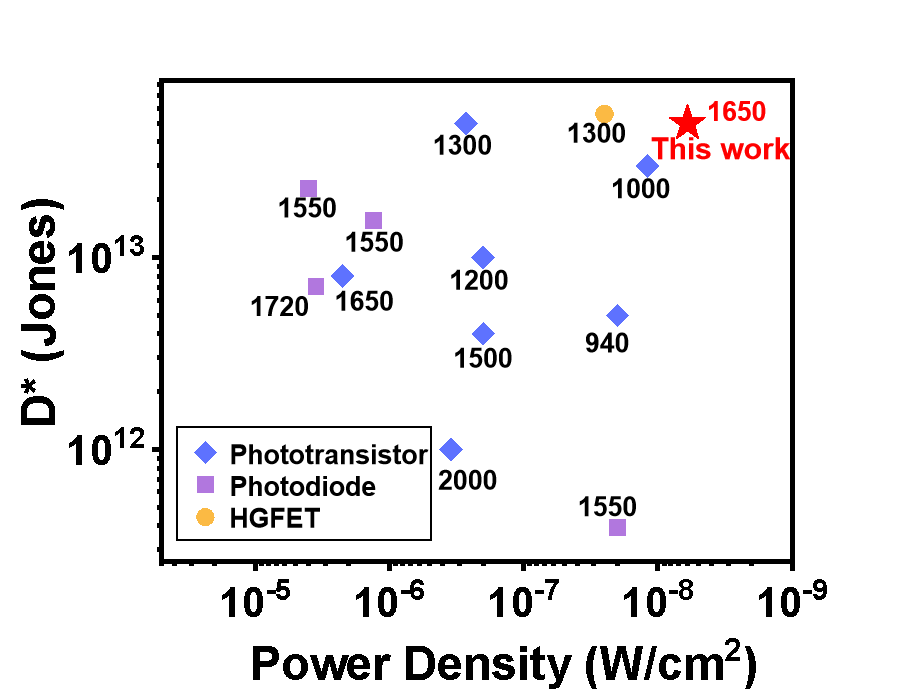


**Figure S17. Performance benchmarking.** Comparison of the champion CNT HGFET’s detectivity and detectable power density with state-of-the-art SWIR photodetectors reported in the literature. ^(2-13)^

Table S3 provides a multi-parameter comparison of representative infrared photodetectors in terms of detectivity, response speed, dark current, and monolithic three-dimensional (M3D) integration capability. As shown, HGFET photodetectors exhibit a distinct advantage in high detectivity under low-light conditions, together with compatibility with direct M3D integration on silicon CMOS readout circuits, which is favorable for low-cost and large-area focal plane array implementation. These characteristics make HGFETs particularly suitable for night-vision and imaging applications. In contrast, due to their relatively slower temporal response, the current HGFET architecture is not optimized for high-speed photodetection applications such as optical communications, where sub-microsecond or nanosecond response times are required.

**Table S3. Comparison of various parameters of HGFETs with other reported infrared detectors.** Note: M3D integration refers to monolithic three-dimensional integration.

| **Material** | **Architecture** | **λ (nm)** | **I_dark_ (A/cm^2^)** | **R (A/W)** | **D* (Jones)** | **Response time (s)** | **M3D integration** | **Ref** |
| --- | --- | --- | --- | --- | --- | --- | --- | --- |
| PbS CQDs | Photodiode | 1550 | 1×10^-10^ | - | 1×10^11^ | 3×10^-7^ | YES | (14) |
| GR/ PbS CQDs | Phototransistor | 950 | - | 4×10^7^ | 7×10^13^ | 1×10^-2^ | YES | (15) |
| GR / Au QDs | Phototransistor | 1550 | - | 83 | 1×10^8^ | 6×10^-7^ | YES | (16) |
| PbS-CQD/Graphene | Phototransistor | 1600 | - | 2×10^6^ | 4×10^12^ | 2×10^-4^ | YES | (17) |
| Si / PbS CQDs | Phototransistor | 1500 | - | 1×10^3^ | 2×10^12^ | 4×10^-4^ | YES | (18) |
| PbS CQDs/IGZO | Phototransistor | 1064 | 3×10^-5^ | 45 | 9×10^12^ | 3×10^-4^ | YES | (19) |
| HgTe CQDs | Phototransistor | 1600 | - | 6×10^-1^ | 4×10^10^ | 1×10^-5^ | YES | (20) |
| PbSe-CQD | Photodiode | 1400 | 1×10^-7^ | 1 | 2×10^12^ | 4×10^-4^ | YES | (21) |
| PbS-CQD | Photodiode | 2100 | 1×10^-5^ | 2×10^-1^ | 6×10^10^ | 7×10^-5^ | YES | (22) |
| WSe_2_​ | Photodiode | 1600 | - | 9 | 3×10^12^ | 4×10^-1^ | YES | (23) |
| OPD | Photodiode | 1060 | 1×10^-9^ | 4×10^-1^ | 2×10^13^ | 1×10^-5^ | YES | (24) |
| GaSe/Ge | Photodiode | 1550 | - | 43 | 1×10^10^ | 1×10^-5^ | NO | (25) |
| Ag_2_Te | Photodiode | 1500 | 2×10^-6^ | 4×10^-1^ | 1×10^12^ | 1×10^-6^ | YES | (26) |
| InGaAs | Photodiode | 1550 | 3×10^-8^ | 1.2 | 1×10^12^ | 1×10^-9^ | NO | (27) |
| PbS CQDs / CNTs | HGFET | **1650** | 1×10^-5^ | **8581** | **5.7×10^13^** | 1×10^-4^ | **YES** | **This work** |


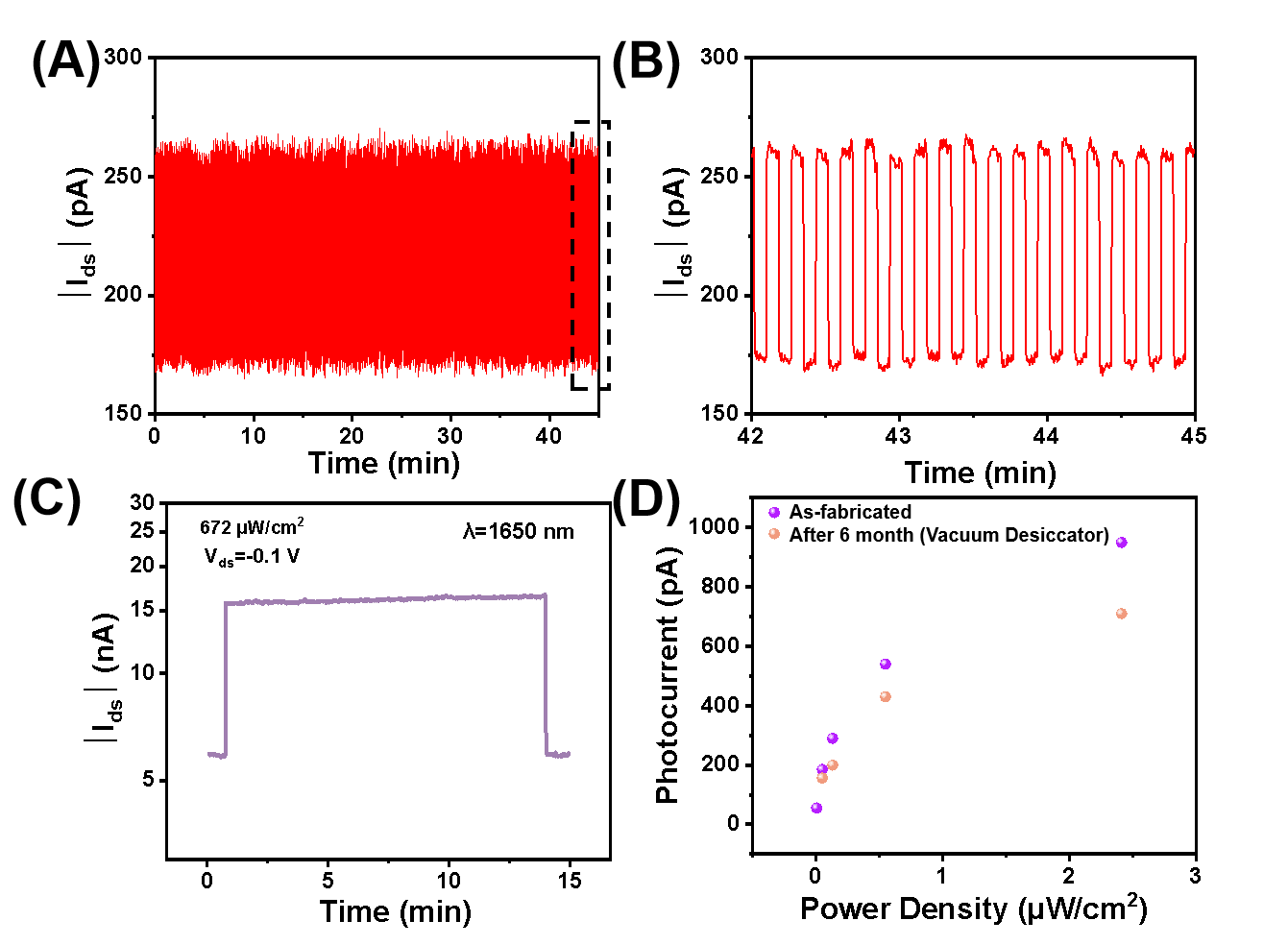


**Figure S18. Operational stability of the fabricated HGFET.** (A) Cyclic on/off switching measurements performed under 940 nm illumination with an intensity of 5 mW cm^-2^ for a total duration of 40 minutes, corresponding to 250 switching cycles. (B) Enlarged view of the cyclic on/off switching behavior highlighted by the dashed box in panel (A). (C) Continuous illumination measurements carried out for approximately 12 min. Due to constraints of the available laboratory conditions, extended light stress tests over tens of hours were not conducted. (D) Photocurrent as a function of incident power density for the as-fabricated HGFET and the same device after 6 months (stored in a vacuum chamber, vacuum level ~1kPa).

The apparent difference in signal smoothness between the cyclic switching test (Figure S18B) and the continuous stability test (Figure S18C) arises from the distinct bandwidths and data acquisition mechanisms of the two measurement systems. The continuous illumination stability (Figure S18C) was recorded using a Keithley 4200-SCS semiconductor parameter analyzer. This instrument employs an integrating analog-to-digital converter with an integration time typically set to $\ge$ 1 power line cycle (NPLC, ~20 ms). This integration process effectively functions as a low-pass filter, averaging out high-frequency noise and resulting in smooth current traces. In contrast, the cyclic on/off switching measurements (Figure S18B) were performed using a high-speed oscilloscope (Keysight HD3) coupled with a low-noise current preamplifier (Stanford Research Systems SR570). To accurately capture the transient response edges, the preamplifier was operated in a high-bandwidth mode. Since the input RMS noise scales with the square root of the measurement bandwidth (Noise ∝ $\sqrt{\mathrm{BW}}$), the wide bandwidth required for high-speed measurements inevitably introduces a higher noise floor, manifesting as minor fluctuations or "burrs" on the signal plateaus. These features are attributed to the measurement setup characteristics rather than the intrinsic instability of the device.

**References**

1. Siegman, A. E., Sasnett, M. W., Johnston, T. F. Choice of clip levels for beam width measurements using knife-edge techniques. *IEEE journal of quantum electronics*. 1991;27:1098-104.

2. Huo, N., Gupta, S., Konstantatos, G. MoS2–HgTe Quantum Dot Hybrid Photodetectors beyond 2 µm. *Advanced Materials*. 2017;29:1606576.

3. Kim, H., Wu, Z., Eedugurala, N., Azoulay, J. D., Ng, T. N. Solution-Processed Phototransistors Combining Organic Absorber and Charge Transporting Oxide for Visible to Infrared Light Detection. *ACS applied materials & interfaces*. 2019;11:36880-5.

4. Jiang, Z., Gao, Y., Hui, W., et al. Self-Assembly PbS Quantum Dot-Conjugated Polymer Hybrid-Layered Phototransistor Enables SWIR Photodetection with High Detectivity. *Advanced Optical Materials*. 2024;12:2303188.

5. Luo, P., Zhuge, F., Wang, F., et al. PbSe Quantum Dots Sensitized High-Mobility Bi2O2Se Nanosheets for High-Performance and Broadband Photodetection Beyond 2 μm. *ACS Nano*. 2019;13:9028-37.

6. Pradhan, A., Roy, A., Tripathi, S., et al. Ultra-high sensitivity infra-red detection and temperature effects in a graphene–tellurium nanowire binary hybrid. *Nanoscale*. 2017;9:9284-90.

7. Wu, D., Guo, J., Du, J., et al. Highly Polarization-Sensitive, Broadband, Self-Powered Photodetector Based on Graphene/PdSe2/Germanium Heterojunction. *ACS Nano*. 2019;13:9907-17.

8. Yang, J., Hu, H., Lv, Y., et al. Ligand-Engineered HgTe Colloidal Quantum Dot Solids for Infrared Photodetectors. *Nano Letters*. 2022;22:3465-72.

9. Liu, J., Liu, P., Chen, D., et al. A near-infrared colloidal quantum dot imager with monolithically integrated readout circuitry. *Nature Electronics*. 2022;5:443-51.

10. Chen, D., Liu, Y., Xia, B., et al. Passivating {100} Facets of PbS Colloidal Quantum Dots via Perovskite Bridges for Sensitive and Stable Infrared Photodiodes. *Advanced Functional Materials*. 2023;33:2210158.

11. Zhou, S., Zhang, X., Wang, Y., et al. Opto-Electrical Decoupled Phototransistor for Starlight Detection. *Advanced Materials*. 2025;37:2413247.

12. Zhou, S. Y., Wang, Y., Deng, C. J., et al. Highly sensitive SWIR photodetector using carbon nanotube thin film transistor gated by quantum dots heterojunction. *Applied Physics Letters*. 2022;120:193103.

13. Liu, Y., Liu, J., Deng, C., et al. Planar Cation Passivation on Colloidal Quantum Dots Enables High-Performance 0.35–1.8 µm Broadband TFT Imager. *Advanced Materials*. 2024;36:2313811.

14. Clifford, J. P., Konstantatos, G., Johnston, K. W., et al. Fast, sensitive and spectrally tuneable colloidal-quantum-dot photodetectors. *Nature Nanotechnology*. 2009;4:40-4.

15. Konstantatos, G., Badioli, M., Gaudreau, L., et al. Hybrid graphene–quantum dot phototransistors with ultrahigh gain. *Nature Nanotechnology*. 2012;7:363-8.

16. Chen, Z., Li, X., Wang, J., et al. Synergistic Effects of Plasmonics and Electron Trapping in Graphene Short-Wave Infrared Photodetectors with Ultrahigh Responsivity. *ACS Nano*. 2017;11:430-7.

17. Nikitskiy, I., Goossens, S., Kufer, D., et al. Integrating an electrically active colloidal quantum dot photodiode with a graphene phototransistor. *Nature Communications*. 2016;7:11954-.

18. Adinolfi, V., Sargent, E. H. Photovoltage field-effect transistors. *Nature*. 2017;542:324.

19. Zhang, C., Yin, X., Qian, G., et al. Gate Voltage Adjusting PbS‐I Quantum‐Dot‐Sensitized InGaZnO Hybrid Phototransistor with High‐Sensitivity. *Advanced Functional Materials*. 2024;34.

20. Chen, M., Lu, H., Abdelazim, N. M., et al. Mercury Telluride Quantum Dot Based Phototransistor Enabling High-Sensitivity Room-Temperature Photodetection at 2000 nm. *ACS Nano*. 2017;11:5614-22.

21. Zhu, T., Zheng, L., Yao, X., et al. Ultrasensitive Solution-Processed Broadband PbSe Photodetectors through Photomultiplication Effect. *ACS applied materials & interfaces*. 2019;11:9205-12.

22. Dong, C., Liu, S., Barange, N., et al. Long-Wavelength Lead Sulfide Quantum Dots Sensing up to 2600 nm for Short-Wavelength Infrared Photodetectors. *ACS applied materials & interfaces*. 2019;11:44451-7.

23. Liu, H., kang, Y., Zhang, L., et al. Exceptionally Low‐Noise Room‐Temperature Shortwave Infrared Photodetector Based on Low‐Frequency Charge Transfer Transition. *Advanced Functional Materials*. 2024;34.

24. Xu, C., Liu, P., Feng, C., He, Z., Cao, Y. Organic photodetectors with high detectivity for broadband detection covering UV-vis-NIR. *Journal of materials chemistry C, Materials for optical and electronic devices*. 2022;1:5787-96.

25. Wang, R., An, Y., Wu, S., et al. A Two-Terminal p-GaSe/n-Ge/Au Heterojunction Phototransistor With a Schottky Barrier Collector for Extension of Short Wavelength Infrared Band. *IEEE transactions on electron devices*. 2025;72:3674-9.

26. Wang, Y., Peng, L., Schreier, J., et al. Silver telluride colloidal quantum dot infrared photodetectors and image sensors. *Nature Photonics*. 2024;18:236-42.

27. Thorlabs. InGaAs photodiode 800-1700 nm FGA015 <https://www.thorlabschina.cn/thorproduct.cfm?partnumber=FGA015>. [
